# Improved 2-pyridyl reductive homocoupling reaction using biorenewable solvent Cyrene™ (dihydrolevoglucosenone)†

**DOI:** 10.1039/d3su00005b

**Published:** 2023-07-17

**Authors:** Daniel A. Webb, Zeid Alsudani, Guolin Xu, Peng Gao, Leggy A. Arnold

**Affiliations:** a Department of Chemistry and Biochemistry and the Milwaukee Institute for Drug Discovery, University of Wisconsin-Milwaukee Milwaukee Wisconsin 53211 USA arnold2@uwm.edu; b Sigma-Aldrich Co. LLC 6000 N. Teutonia Ave. Milwaukee WI 53209 USA

## Abstract

The synthesis of 5,5′-bis(trifluoromethyl)-2,2′-bipyridine using 2-bromo-5-(trifluoromethyl) pyridine was achieved at 50 °C using palladium acetate, tetrabutylammonium iodide (TBAI), potassium carbonate, and isopropanol in Cyrene™ (dihydrolevoglucosenone), a bio-renewable “green” solvent formed by a two-step process from cellulose. Improvements were achieved with 50% of γ-valerolactone (GVL) in Cyrene™ resulting in a 95% yield and 99% product purity without the use of column chromatography or recrystallization. At 80 °C, the reaction was completed within 1 h. Full conversion with 1 mol% instead of 15 mol% of palladium acetate was observed within 10 h. We showed that the formed 2,2′-bipyridine product significantly accelerated the reaction probably due to the stabilization of the catalytic species. The addition of TBAI was essential for the rapid homocoupling, however, 20 mol% of TBAI was sufficient to reach full conversion of 2-bromo-5-(trifluoromethyl) pyridine within 6 h at 80 °C. Another improvement was observed with the substitution of isopropanol by 1,4-butanediol achieving full conversion within 6 h. 2-Bromopyridines with electron withdrawing substituents in the 6, 5, 4 ring position reacted under these conditions. 2-Bromopyridines with an electron donating substituent reacted slower. Overall, we demonstrated that the 50% GVL in Cyrene™ blend is a superior “green” and less toxic alternative to dimethylformamide for the reductive homocoupling reaction. Using a quantitative scoring for twelve principles of green chemistry (DOZN™), we found significant improvements that were mediated by higher yield (atom economy), shorter heating time and lower reaction temperature (energy efficiency), safer solvent (hazardous chemical synthesis), and safer chemistry (accident prevention).

## Abstract 2

The synthesis of 5,5′-bis(trifluoromethyl)-2,2′-bipyridine was achieved using a palladium catalyzed homocoupling reaction with reductant isopropanol in a bio-renewable “green” solvent blend of Cyrene™ and γ-valerolactone.

## Graphical abstract

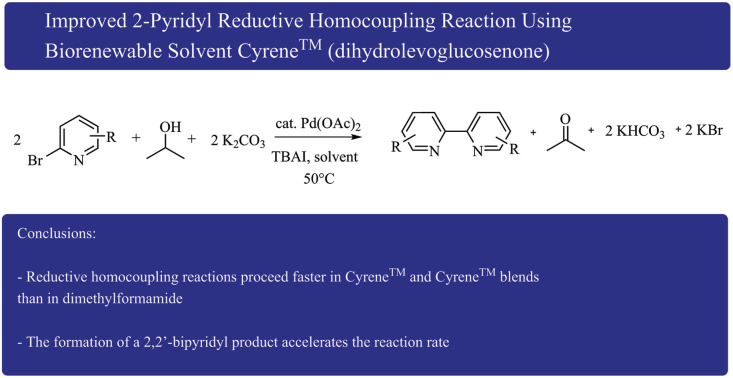

**Sustainability spotlight**

The application of biorenewable materials for the production of chemicals is an important step to reduce the use of products derived from fossil fuels. Carbon containing resources such as natural gas and crude oil are limited global resources. Furthermore, the extraction and processing of these resources are causing a significant risk to the population and environment. Our work is focused on the application of biorenewable solvent Cyrene™, a renewable, nontoxic, and cheap material derived from cellulose. Solvents are usually used in larger quantities than reactants to support chemical reactions, thus the identification of new biorenewable and nontoxic alternatives is extremely important for a sustainable future of chemistry. We proposed to use Cyrene™ as an alternative polar aprotic solvent. This class of solvents are used very frequently for chemical processes. Members of this solvent class are dimethylformamide and acetonitrile, which cause acute and chronic health effects and harm the environment. Remarkably, we found that Cyrene™ significantly reduced the reaction time of reductive homocoupling when used as solvent blend with γ-valerolactone, another biorenewable solvent. Furthermore, the reaction could be carried out at a lower reaction temperature reducing the amount of energy used for the synthesis of bipyridine compounds. The following UN sustainable development goals are reflecting in this work: good health and well-being (SDG 3), clean water and sanitation (SDG 6), industry, innovation, and infrastructure (SDG 9), climate action (SDG 13), life below water and on land (SDG 14 & 15).

## Introduction

Non-toxic and renewable solvents are very attractive to produce small molecules commercially and in the research laboratory. Greener alternatives to dimethylformamide (DMF), a commonly used polar aprotic organic solvent appreciated for its ability to dissolve organic compounds while being water soluble has led to the discovery of alternatives such as Cyrene™ (dihydrolevoglucosenone).^1^ Cyrene™ is a bio-based solvent that is derived from a two-step process beginning with cellulose.^2^ The cost of this solvent is comparable with DMF, but it can be anticipated that the price will decline with further demand. An important advantage is that Cyrene™ is non-toxic and biodegradable.^3^ Menschutkin reactions and fluorinations have been carried out in Cyrene™ achieving better reaction rates than standard aprotic polar solvents.^1^ Interestingly, the viscosity of Cyrene™ allowed for great improvements in the process of graphene dispersion.^4^ Other reactions have been reported using Cyrene™ as a solvent for example peptide coupling reactions using HATU,^5^ an amide forming reaction with acid chlorides,^6^ reactions that formed urea products,^7^*N*-alkylations,^8^ S_N_Ar reactions,^8^ and aldimine reactions.^8^ Some transition metal-catalyzed reactions in Cyrene™ have been reported. These include a Suzuki–Miyarua reaction^9^ and a Sonogashira and Cacchi-type annulation reaction.^10^ Importantly, the application of Cyrene™ significantly increases the sustainability of the production of chemicals and materials.

**Scheme 1:**
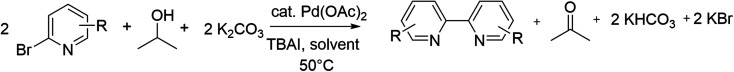
Reductive homocoupling using isopropanol as reductant.

Herein, we report the use of Cyrene™ and Cyrene™ solvent blends to produce substituted 2,2′-bipyridines *via* a reductive homocoupling reaction (Scheme 1).

Starting from aryl halides, this reaction is usually catalyzed by palladium in the presence of another metal or a compound that is oxidized in the process.^11^ Only a limited number of palladium-catalyzed reductive homocoupling reactions using non-toxic alcohols as reductants have been reported for the conversion of iodo- and bromopyridines. Zeng *et al.* reported the used of 3-pentanol as solvent using 3 mol% Pd(dppf)Cl_2_ and 7.5 equivalent of CaF as base.^12^ The reaction proceeded at 100 °C for bromo- and iodobenzene as well as 2-iodo and 2-bromopyridine. The use of 1,4-butanediol as solvent was reported by Huang *et al.* in conjunction with 0.5 mol% Pd(OAc)_2_, 1.1 equivalent of cesium carbonate at 75 °C proceeding for a wide range of substituted aryl iodides, iodopyridines, and iodothiophenes.^13^ The reaction times varied between 2 to 24 h and resulted in yields between 39–95%. Other green synthetic approaches for homocoupling and Ullmann reactions have been reported.^14^ This includes several reports using water as solvent. Gädda *et al.* introduced a homocoupling of aryl iodides using microwave irradiation with 4.8 mol% of Pd/C at 150 °C.^15^ The conversion for 2-iodopyridine was 47%. Other catalysts introduced included amphiphilic Pd carbon spheres giving conversions between 44–53% with a catalyst loading of 0.5% at 90 °C.^16^ This work was followed by others, reporting Pd and Au nanosphere catalysts resulting in higher biaryl yields with the use of aryl bromides^17,18^ and aryl chlorides^19^ using high temperatures and in some instances long reaction times.^14^ Other approaches include the use of aryl boronic acids in combination with copper(i)chloride, a water extract of pomegranate ash (WEPA) and ethanol at room temperature^20^ or Pd-catalyzed reductive homocouplings with water extracted papaya bark ash and tobacco using ethanol or DMF as co-solvents.^21,22^ These reports cited many other green approaches for the homocoupling of aryl boronic acids, which are usually synthesized from aryl halides. Recently, the application of a recyclable glycerol-based deep eutectic solvent was reported for the homocoupling of (hetero)aryl chlorides at 80 °C within 12 hours.^23^

## Experimental

### Materials

All reagents and solvents^24^ were obtained from Sigma-Aldrich Co. LLC, unless otherwise noted, and used as received.

### Analytical procedure

100 μL aliquots were taken from the reaction at the desired time points and added to a Pasteur pipette packed with cotton and 300 mg of Celite 545. The Celite was washed with 1 mL of dichloromethane. 25 μL of the collected filtrate was added to 1 mL of HPLC grade methanol and analyzed by HPLC (Shimadzu Nexera series) with a Photo Diode Array detector (PDA, Shimadzu SPD-M30A) and a single quadrupole mass analyzer (LCMS 2020, Shimadzu, Kyoto, Japan). 0.1% formic acid in methanol and 0.1% formic acid in water was used as the mobile phase. Analytes were separated using a Restek Pinnacle-C18 (4.6 mm × 50 mm, 5 μm particle size) column with gradient elution at a flow rate of 0.5 mL min^−1^. The time program of mobile phase was 20% to 100% over 20 min followed by 3 min at 100%. The column was equilibrated to 20% methanol in water for each injection. Data was acquired by PDA having a range of 190–700 nm but using a wavelength of 264 nm for quantification. Interface temperature, desolvation line temperature and heat block temperature were 350 °C, 250 °C, and 400 °C, respectively. Nebulizing gas flow was 1.5 L min^−1^ and drying gas flow was 13 L min^−1^. Relative reaction conversion percentage was determined by the ratio of peak area of starting material to product.

### Standard procedure

To a 20 mL Radley reaction tube was added 12 mL of solvent. The solvent was degassed by bubbling nitrogen through the solvent for 15 min. 2-Bromo-5-(trifluoromethyl)pyridine (0.75 g, 3.32 mmol) was added, followed by the addition of Pd(OAc)_2_ (112.5 mg, 0.5 mmol, 15 mol%), tetrabutylammonium iodide (1.50 g, 4.06 mmol), and potassium carbonate (0.75 g, 5.43 mmol). The reaction tube was capped and heated to 50 °C. The desired temperature was reached within 15 min. After 30 min isopropyl alcohol (0.56 mL, 7.32 mmol) was added *via* syringe and the reaction was stirred for the desired amount of time. Aliquots were taken at the desired time points. Upon completion of the reaction by TLC (30% EtOAc in hexanes) the reaction mixture was filtered through Celite and washed with 15 mL Cyrene™/GVL. The mixture was extracted with heptane (25 mL, 6×). The fractions were combined and washed with water (150 mL) to remove any residual solvent. The organic layer was dried with MgSO_4_ and concentrated to dryness to yield up to 95% of 5,5′-bis(trifluoromethyl)-2,2′-bipyridine as a white solid (99% purity). ^1^H NMR (500 MHz, CDCl_3_) *δ* 8.97 (m, 2H), 8.64–8.62 (m, 2H), 8.11–8.09 (m, 2H); ^13^C NMR (126 MHz, CDCl_3_) *δ* 157.68, 146.30 (q, ^3^*J*_CF_ = 4.05 Hz), 134.29 (q, ^3^*J*_CF_ = 3.49 Hz), 127.14 (q, ^2^*J*_CF_ = 33.02 Hz), 123.50 (q, ^1^*J*_CF_ = 272.29 Hz), 121.24; ^19^F NMR (471 MHz, CDCl_3_) *δ* −62.53.

## Results and discussion

The initial reductive homocoupling of 2-bromo-5-(trifluoromethyl)pyridine was carried out with 15 mol% Pd(OAc)_2_, 1.2 equivalents of tetrabutylammonium iodide (TBAI), 1.6 equivalents of potassium carbonate, and 2.2 equivalents of isopropanol (IPA) in different solvents at 50 °C (Table 1).

**Table 1:** Reductive homocoupling of 2-bromo-5-(trifluoromethyl)pyridine in different solvents

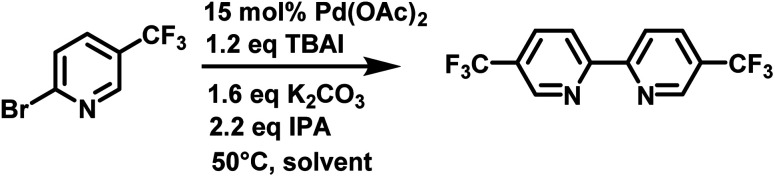			
Solvent	Conversion^a^ after 24 h (%)	cEF (complete *E* factor) (kg kg^−1^)	DOZN score
Cyrene™	98	49.5	8
Dimethylformamide (DMF)	53	76.4	21
Acetonitrile (ACN)	59	61.0	18
γ-Valerolactone (GVL)	53	82.0	15
2-Methyltetrahydrofuran	23	166.4	32
*N*-Methylpyrrolidone	16	270.3	39
1-Butanol	11	337.9	40
Dimethoxyethane (DME)	7	554.6	43
Water	0	∞	—

a Reductive homocoupling of 2-bromo-5-(trifluoromethyl)pyridine. All reactions were conducted in 12 mL of indicated solvent (276 mM) at 50 °C. Aliquots were taken at the desired time points and analyzed by reverse phase HPLC at 264 nm using a C18 column. Reaction conversion percentage was determined by the ratio of peak area of starting material to product.

Reactions with commonly used solvents were conducted with Cyrene™, DMF, acetonitrile (ACN), γ-valerolactone (GVL), 2-methyltetrahydrofuran (2-MeTHF), *N*-methylpyrrolidone (NMP), 1-butanol, dimethoxyethane (DME), and water. For details see the standard analytical procedure. Cyrene™ was by far the best solvent for this reaction with a conversion of 98% after 24 h. DMF, which has shown to be a reproductive toxin,^25^ resulted in a slow reaction achieving 53% in that same time period. Similar conversions were observed with ACN, which is not considered a green solvent, and GVL. GVL is a green solvent that has recently become useful as an alternative to DMF and NMP.^26^ We also tested 2-MeTHF, a green solvent made from furfural, which in turn can be isolated from biomass by distillation under acidic conditions.^27^ The homocoupling reaction in 2-MeTHF showed 23% conversion after 24 h. The homocoupling in 1-butanol and DME after 24 h reached a conversion of 11% and 7%, respectively. The starting materials were not soluble in water thus, no conversion was observed.

We calculated the complete *E* factor (cEF) for these solvents.^28^ The production of 5,5′-bis(trifluoromethyl)-2,2′-bipyridine in Cyrene™ with an cEF of 49.5 kg kg^−1^ still falls into the category of comparable processes to synthesize fine chemicals (5–50 kg kg^−1^). Other solvents generate more waste per product due to the lower conversion. These cEF might even be higher due to incomplete reaction that can complicate product purification and might require additional amounts of solvent. In addition, we used the DOZN™ quantitative scoring online analysis to evaluated the different solvents for this reaction.^29^ In addition to the mass analysis, this green chemistry tool takes into account safety, hazards, energy efficiency, renewability, and pollution. The aggregate DOZN™ scores depend on the atom economy and are therefore following the trend of the cEF. However, the conversion of starting material is identical for solvents GVL and DMF but the DOZN™ scores are different (15 *vs.* 21, Table 1) because GVL is a renewable solvent, non-toxic and safer to use.

**Fig. 1:**
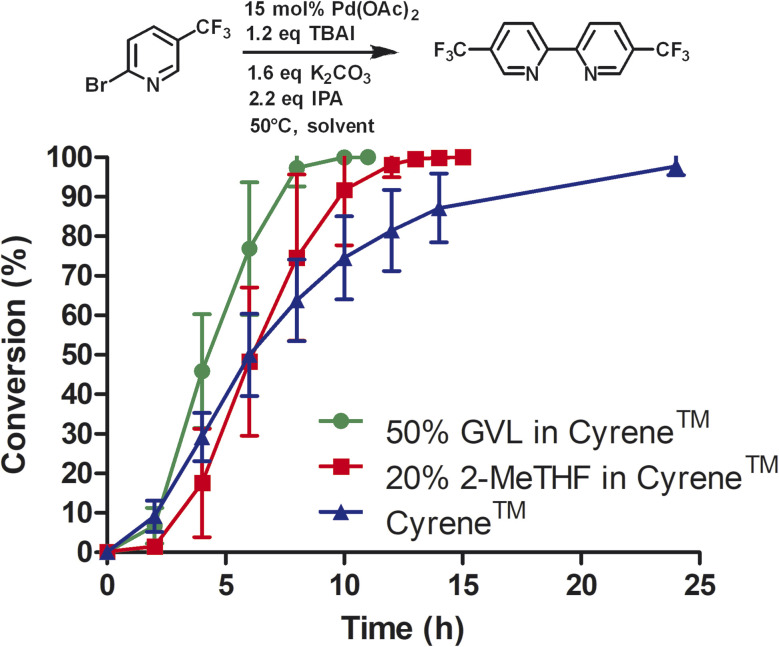
Reductive homocoupling of 2-bromo-5-(trifluoromethyl)pyridine. All reactions were conducted in 12 mL of indicated solvent (276 mM) at 50 °C. Aliquots were taken at the desired time points and analyzed by reverse phase HPLC at 264 nm using a C18 column. Reaction conversion percentage was determined by the ratio of peak area of starting material to product.

Next, we compared Cyrene™ and commercially available Cyrene™ blends that consisted of 50% GVL in Cyrene™ and 20% 2-MeTHF in Cyrene™ (Fig. 1).

Three independent trials for each solvent type were conducted. Cyrene™ itself significantly improved the rate of the reaction. In contrast to DMF, the reaction reached full conversion after 24 h. Interestingly, Cyrene™ blends with GVL and 2-MeTHF further accelerated the homocoupling of 2-bromo-5′-(trifluoromethyl)pyridine. For some trials with Cyrene™/2-MeTHF, 100% conversion was observed after 10 h and for some trials with Cyrene™/GVL the reaction was complete within 8 h. All reactions were slow during the first 2 h and accelerated considerably after that. For solvents that did not contain Cyrene™, the initial reaction rate was significantly slower. The isolated yields for reactions carried out in Cyrene™ and Cyrene™ blends were up to 95%. Also, simple extraction with heptane provided pure 5,5′-bis(trifluoromethyl)-2,2′-bipyridine as product (99% pure by HPLC). Other homocoupling reactions *e.g.* using solvent pentanol reported the formation of almost 20% of pyridine as dehalogenation product.^30^ For these reactions further purification was employed such as recrystallization or column chromatography, which required a significant volume of solvents. In the case of column chromatography using hexanes and ethyl acetate, azeotropes are obtained that prevent recycling by simple distillation.^31^ Heptane used for the extraction of 5,5′-bis(trifluoromethyl)-2,2′-bipyridine was reused as extractant after the evaporation to improve the sustainability of the reaction. We were not able to recover Cyrene™ or Cyrene™ blends because of their high boiling points.

**Fig. 2:**
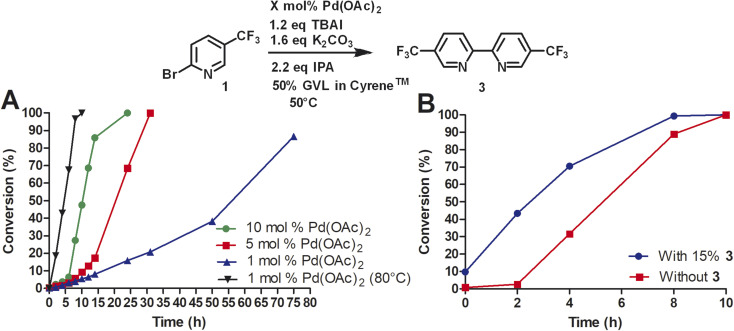
(A) Reductive homocoupling of 1 in the presence of 1, 5, and 10 mol% of Pd(OAc)_2_; (B) reductive homocoupling of 1 in the presence of 15 mol% of Pd(OAc)_2_ with and without the addition of 10% of 5,5′-bis(trifluoromethyl)-2,2′-bipyridine. All reactions were conducted with 12 mL of indicated solvent. Aliquots were taken at the desired time points and analyzed by reverse phase HPLC at 264 nm using a C18 column. Reaction conversion percentage was determined by the ratio of peak area of starting material to product.

Due to the high cost of Pd(OAc)_2_ and relatively high catalyst loading (15 mol%), we examined the effect of lower catalyst loading in respect to conversion (Fig. 2A). The standard procedure using 15 mol% palladium acetate at 50 °C afforded full conversion using the Cyrene™/GVL blend in proximately 8 h. Using 10 mol% Pd(OAc)_2_, the reaction reached full conversion at 24 h. 27.4% conversion was observed at 8 h. The use of 5 mol% Pd(OAc)_2_ resulted in full conversion at 31 h. 5.7% conversion was observed at 8 h. The homocoupling reaction employing 1 mol% Pd(OAc)_2_ reached 87% conversion after 75 h. 6.4% conversion was observed at 12 h. The increase of the reaction temperature to 80 °C accelerate the reaction rate significantly achieving full conversion within 10 h. Thus, a lower catalyst loading could be employed but required a higher reaction temperature to reach completion in a reasonable amount of time. At 50 °C, a pronounced slow onset of the reaction was observed, possibly due to low solubility or slow formation of the catalytic species. 2,2′-Bipyridines have been widely used as ligands for palladium-catalyzed reactions^32^ and therefore we explored the addition of product 3 at the start of the reaction (Fig. 2B). We observed a faster onset of the reaction with the addition of 3 than without. The graph begins with 10% conversion because compound 3 is also the corresponding product. Overall, we found that the reaction with the addition of 3 serving as palladium ligand was two hours faster than the reaction without.

**Fig. 3:**
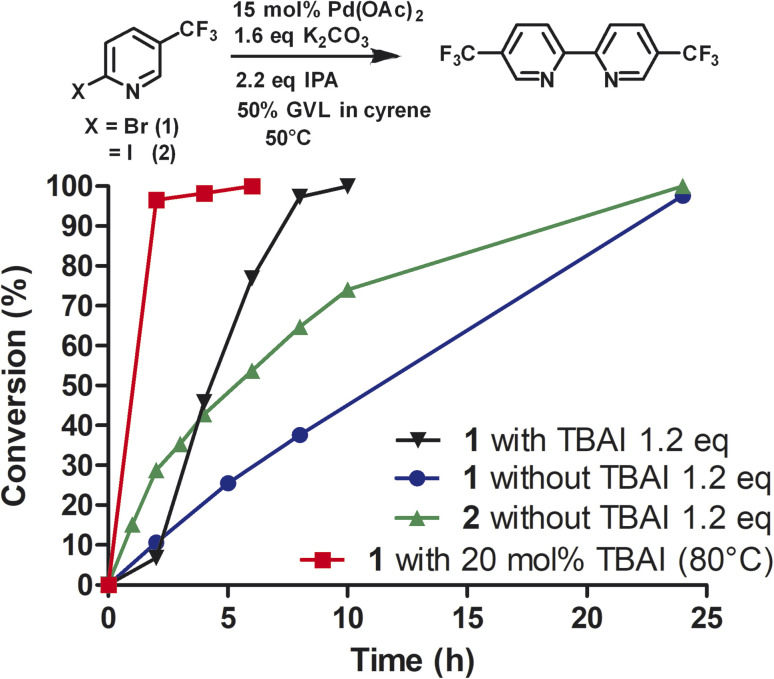
Reductive homocoupling of 1 and 2 in the presence and absence of TBAI. All reactions were conducted with 12 mL of 50% GVL in Cyrene™ (276 mM). Aliquots were taken at the desired time points and analyzed by reverse phase HPLC at 264 nm using a C18 column. Reaction conversion percentage was determined by the ratio of peak area of starting material to product.

To investigate the role of TBAI, which was added to assist in an assumed Finkelstein type reaction (bromo-iodo exchange reaction), we carried out the homocoupling of 2-bromo-5-(trifluoromethyl)pyridine (1) without TBAI using the Cyrene™/GVL blend as solvent (Fig. 3).

**Scheme 2:**
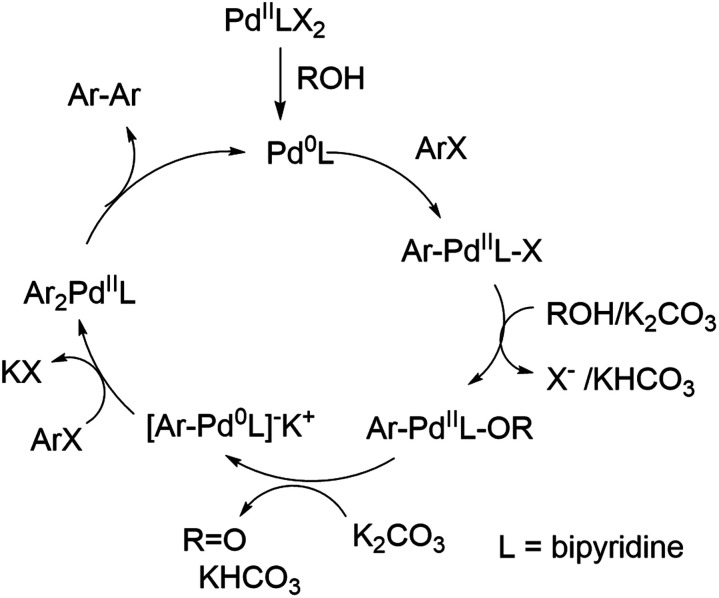
Proposed catalytic cycle of the palladium-catalyzed reductive homocoupling.

In the absence of TBAI, the homocoupling of 1 was slower than with TBAI. A conversion of 97% was achieved after 24 h instead of 8 h with TBAI. Thus, TBAI significantly accelerated the rate of the reaction. Assuming that TBAI will form 2-iodo-5-(trifluoromethyl)pyridine (2) from 1*in situ*, we investigated 2 in the absence of TBAI. During the beginning of the reaction, the conversion of 2 was substantially faster but slowed down after three hours. Full conversion of 2 was observed after 24 h. Thus, it can be concluded that substituted 2-bromopyridines in the presence of TBAI react faster than substituted 2-iodopyridines. During the reaction of 2-bromo-5-(trifluoromethyl)pyridine with TBAI, iodide is generated (Scheme 2). Thus, it can be hypothesized that sub-stoichiometric amounts of TBAI are sufficient to accelerate this reaction. Indeed, we observed that 20 mol% of TBAI at an elevated temperature of 80 °C is sufficient to support 97% conversion of 2-bromo-5-(trifluoromethyl)pyridine within 2 h and full conversion in 6 h.

**Fig. 4:**
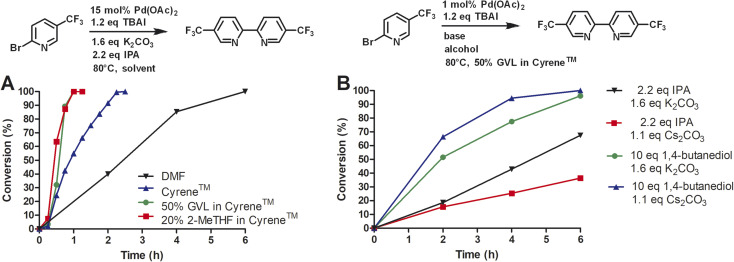
Reductive homocoupling at 80 °C. All reactions were conducted in 12 mL of indicated solvent (276 mM). (A) Investigation of Cyrene™ and Cyrene™ solvent blends; (B) investigation of different alcohols and bases. Aliquots were taken at the desired time points and analyzed by reverse phase HPLC at 264 nm using a C18 column. Reaction conversion percentage was determined by the ratio of peak area of starting material to product.

Next, we investigated the conversion of this homocoupling reaction for Cyrene™ and Cyrene™ blends at 80 °C (Fig. 4A). For the homocoupling reaction carried out in DMF, we observed a significantly faster reaction with complete conversion within 6 h. In comparison, at 50 °C, the conversion in DMF was 3% after 5 h. Similar temperature-dependent accelerations of conversion were observed for Cyrene™ and Cyrene™ blends. For Cyrene™, full conversion was observed after 150 min. For the homocoupling carried out in GVL/Cyrene™ and 2-MeTHF/Cyrene™, full conversion was observed after 60 min. All reactions showed very little conversion during the first 15 min of the reaction. Wilson *et al.* reported that Cyrene™ is stable in the presence of potassium carbonate at 25 °C, whereas at 50 and 100 °C the formation of an aldol product was observed.^10^ We did observe the formation of the Cyrene™ aldol product by thin liquid chromatography and nuclear magnetic resonance, but this side product did not influence the conversion or yield of the reaction.

Huang *et al.* reported a yield of 59% for homocoupling product of 2-iodopyridine using 0.5% of Pd(OAc)_2_, 1.1 equivalents of cesium carbonate, 1,4-butanediol as solvent at 75 °C after 24 h.^13^ Using 2-bromo-5-(trifluoromethyl)pyridine under standard reaction conditions with 1 mol% of Pd(OAc)_2_ at 80 °C, we achieved a yield of 95% after 10 h at 80 °C. However, we investigated a different base and alcohol (Fig. 4B). The change from potassium carbonate (1.6 eq.) to cesium carbonate (1.1 eq.) reduced the reaction rate. Secondly, we substituted isopropanol (2.2 eq.) for 1,4-butanediol (10 eq.) and kept potassium carbonate as base. This combination significantly increased the reaction rate and completion of conversion was observed after 6 h instead of 10 h. When cesium carbonate (1.1 eq.) was used in conjunction with 1,4-butanediol, the reaction rate appeared to increase even more, however, this change might not be statistically different if repeated multiple times. Huang *et al.* proposed that 1,4-butanediol might coordinate as bidentate ligand to palladium and therefore increase the stability of the catalytic species. Although we have no evidence to support this, we concluded that 1,4-butanediol (10 eq.) is superior to isopropanol (2.2 eq.) for our reaction and that potassium carbonate can still be used as a cheap base.

As reported by Hassan *et al.*,^33^ the preparation of biaryls *via* a reductive homocoupling using aryl bromides in DMF with K_2_CO_3_ or Et_3_N as base and the addition of IPA at 115 °C is possible. Therefore, we investigated different substrates with the Cyrene™/GVL blend in the presence of 15 mol% Pd(OAc)_2_, TBAI (1.2 eq.), and IPA (2.2 eq.) at 50 °C (Table 2).

**Table 2:** Reaction progress of reductive homocoupling reactions with various substrates^a^

Entry	Starting material	Conversion (%)				
		4 h	8 h	12 h	24 h	48 h
1	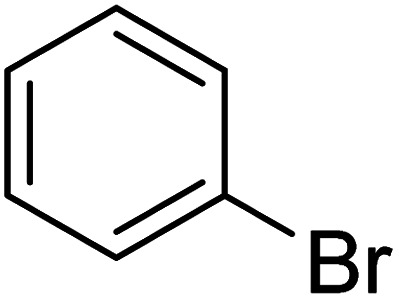	0.0	0.0	0.0	0.0	0.0
2	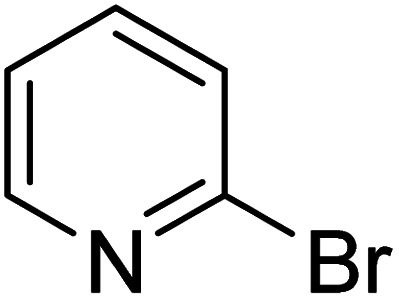	0.0	3.3	23.7	60.9	81.3
3	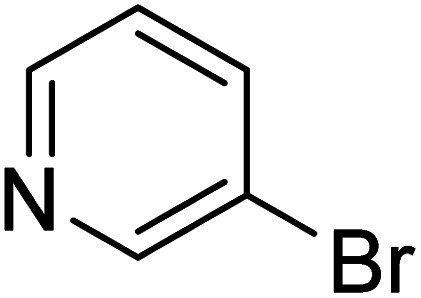	0.0	0.0	0.0	0.0	0.0
4	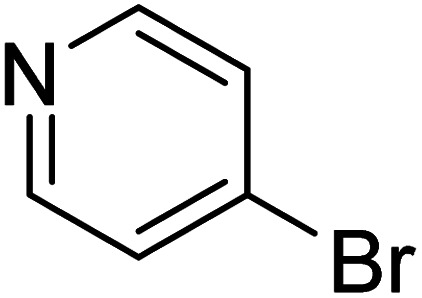	0.0	0.0	0.0	0.0	0.0
5	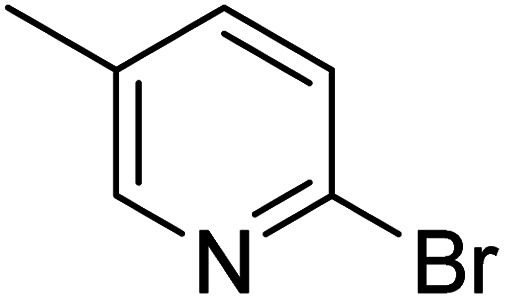	0.0	0.0	0.0	2.0	6.4
6	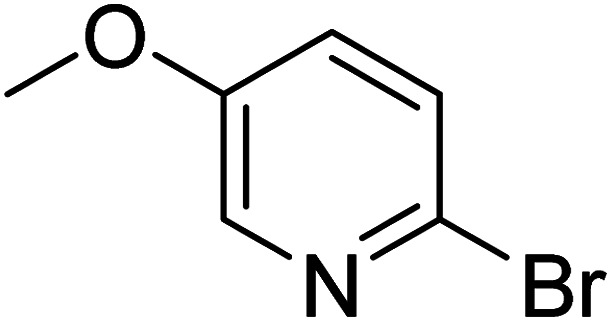	0.0	0.0	0.6	10.4	31.3
7	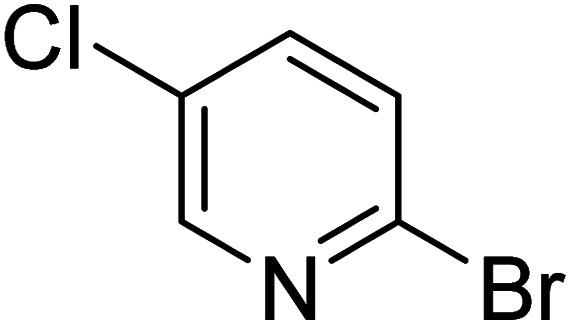	20.3	64.9	72.7	81.4	84.5
8	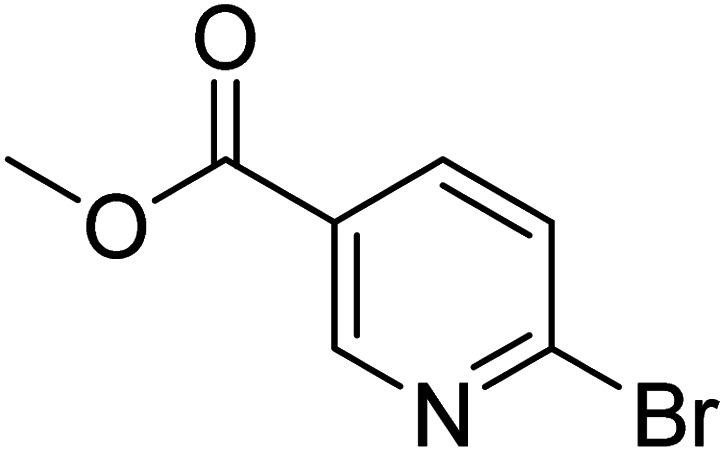	0.0	5.6	37.3	97.2	100
9	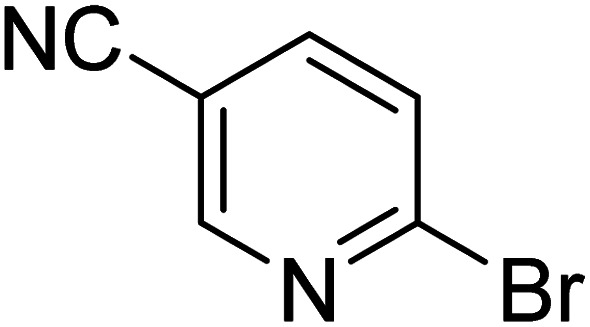	35.5	76.1	100	100	100
10	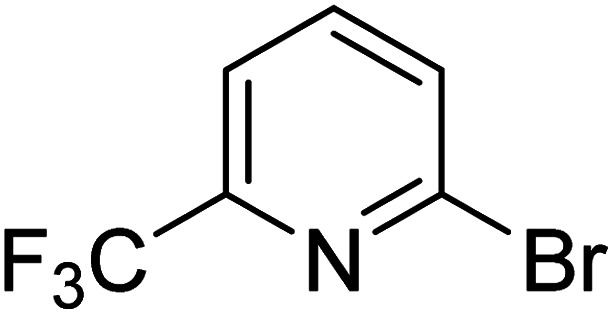	5.5	11.4	16.0	33.6	90.0
11	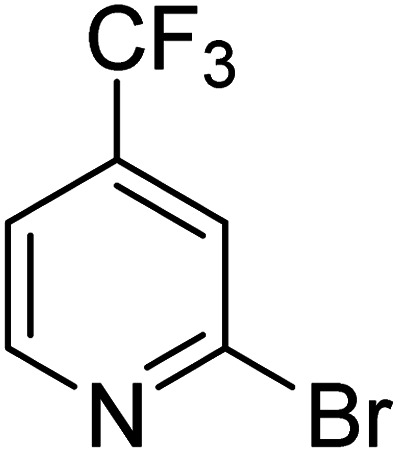	31.7	87.8	100	100	100
12	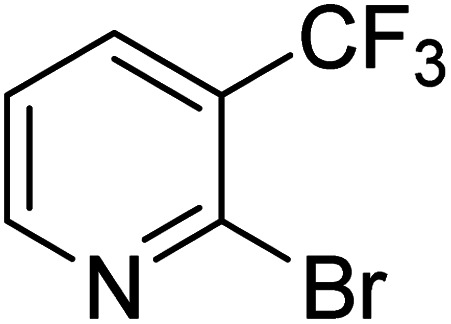	0.0	0.0	<1	<1	<1

a All reactions were carried out with 15 mol% Pd(OAc)_2_, 1.2 equiv. of TBAI, 1.6 equiv. of K_2_CO_3_, and 2.2 equiv. of IPA in 50% GVL in Cyrene™ at 50 °C.

In the case of bromobenzene, we did not observe any conversion (Table 2, entry 1). This result supported our observation that the formation of a bipyridine product is useful to accelerate the homocoupling under these conditions. Next, we investigated 2-bromopyridine (Table 2, entry 2). 81.3% conversion was observed after 48 h. At 8 h, 3.3% conversion was observed, whereas 2-bromo-5′-(trifluoromethyl)pyridine was fully converted at that time point. Thus, the electron withdrawing character of the CF_3_ not only accelerated the initial formation of 3 in contrast to 2-bromopyridine, but also resulted in a faster reaction with the completion within 8 h. Bipyridine ligands are most effective with the position of the nitrogens in the 2,2′ position. This enables bidentate coordination of palladium. To investigate this fact, 3- and 4-bromopyridine were employed under the same reaction conditions (Table 2, entries 3 and 4). Both substrates exhibited no conversion. Next, we investigated different substituents in the 5-position. For the electron donating methyl group, we observed 6.4% conversion of 2-bromo-5-methylpyridine over a period of 48 h (Table 2, entry 5). The methoxy group in *para* position is predominantly electron donating (resonance) and has less inductive electron withdrawing character.^34^ For 2-bromo-4-methoxypyridine, we observed a conversion of 31.3% after 48 h (Table 2, entry 6). Interestingly, the conversion after 24 h was 10.4% but tripled for the next 24 h reaching 31.3% conversion. 2-Bromo-4-chloropyridine converted very quickly during the first 8 h (64.9%) (Table 2, entry 7). However, the reaction rate slowed and reached only 84.5% after 48 h. Esters are electron withdrawing groups and were well tolerated in this homocoupling reaction. Methyl 6-bromopyridine-3-carboxylate was a good substrate for this reaction resulting in full conversion after 48 h (Table 2, entry 8). Also in this case, very little conversion was observed during the first 8 h. The strong electron withdrawing properties of 2-bromo-4-cyanopyridine resulted in a rapid homocoupling reaction with full conversation after 12 h (Table 2, entry 9). Thus overall, the relationship between reaction rate and substitution followed Hammett defined sigma values. Moving the CF_3_ group to the 6 position reduced the reaction rate and resulted in 90% conversion for 2-bromo-6-(trifluoromethyl)pyridine after 48 h (Table 2, entry 10). In contrast, 2-bromo-4-(trifluoromethyl)pyridine was converted almost as quickly as 1, reaching full conversion after 12 h. Although the position of the CF_3_ group is closer to the bromine for both substrates in comparison to 1, the pyridine nitrogen is counteracting the electron withdrawing effect CF_3_ group for entry 10. Probably due to steric effects, 2-bromo-3-(trifluoromethyl)pyridine was not converted under these reaction conditions (Table 2, entry 12). We also employed 2-bromopyridine-5-carbaldehyde but found a very complex mixture of products due to the described formation of aldol products with Cyrene™ (data not shown).^35^

We used the DOZN™ quantitative scoring analysis to evaluate 12 principles of green chemistry for the optimized process using GVL/Cyrene™ at 50 °C for a 12 h reaction time and compared it with the reaction with DMF. The principal analysis showed score differences in the use of less hazardous materials (100.00 *vs.* 22.29), design of safer chemicals (1.19 *vs.* 0.62) and safer solvents and auxiliaries (42.62 *vs.* 10.03). Especially the long reaction time in DMF and lower yield resulted in a high score for design of energy efficiency (96.79 *vs.* 17.33) and inherently safer chemistry of accident prevention (100.00 *vs.* 17.89). The renewable aspect of Cyrene™ further reduced the score for the use of renewable feedstocks from 100.00 to 7.91. Thus, the overall score for the homocoupling in DMF was 456.98 *vs.* 87.18 for the reaction in 50% GVL/Cyrene™. The aggregate score was reduced from 38 to 7 resulting in a total improvement of 82%.

It can be concluded that the 50% GVL/Cyrene™ blend is superior to Cyrene™ and more importantly performed better than other commonly used polar aprotic solvents for the palladium catalyzed reductive homocoupling with isopropanol. It has been reported that the presence of isopropanol is important for the reduction of Pd^II^ to Pd^0^,^12^ which in turn undergoes an oxidative addition of 1 (ArX) (Scheme 2). The bipyridine product/ligand formed during the reaction is likely to stabilize the palladium complex and improves solubility of the catalytic species. The formation of reduced aryl compounds by a dehalogenation side reaction has been reported at higher temperature.^33^ For our reaction conditions, this side reaction was not observed. In contrast, coordination of isopropanol in the presence of potassium carbonate enabled the formation of a Pd^0^–Ar complex and formation of acetone. A second oxidative addition of 1 (ArX) afforded the diarylpalladium complex as proposed by Jutand and Mosleh,^36^ which resulted in the formation of the aryl–aryl bond and regenerates Pd^0^ for the next catalytic cycle. We demonstrated that the addition of 2,2′-bipyridine 3 as a ligand enabled a rapid homocoupling reaction right from the start of the reaction. The addition of 3 might improve the conversion of substrates summarized in Table 1, however, additional purification is necessary to separate the products from 3. An application of a water-soluble 2,2′-bipyridine ligand that is also soluble in the GVL/Cyrene™ blend might overcome this purification challenge. Furthermore, we demonstrated that the reaction is significantly faster at 80 °C and that 1 mol% of Pd(OAc)_2_ can be used. This higher temperature might have enabled full conversion of substrates summarized in Table 1. Huang *et al.* reported that the homocoupling in 1,4-butanediol is significantly faster than in isopropanol and that cesium carbonate is superior to potassium carbonate.^13^ We confirmed this fact using 10 equivalents of 1,4-butanediol and 1.1 equivalents of cesium carbonate for the reaction in 50% GVL/Cyrene™. Although the greenness of the reaction was significantly improved with the use of biorenewable and non-toxic solvent Cyrene™ in addition to a shorter reaction time and lower reaction temperature, there are aspects that can be invested to further improve this reaction. This includes further reduction of the catalyst loading and reaction temperature by using an optimized palladium ligand. The recycling of Cyrene™ could be another improvement, which is often accomplished in industry with a wide variety of solvents. Finally, the ability to generate pure product without column chromatography is an important step to reduce the use of problematic solvents.

## Conflicts of interest

Sigma-Aldrich Company Limited is a subsidiary of Merck KGaA. Sigma-Aldrich provided materials including Cyrene™ and Cyrene™ blends that are commercial products of Sigma-Aldrich. Three authors (Zeid Alsudani, Guolin Xu, and Peng Gao) are employees of Sigma-Aldrich. UWM Ph.D. student Daniel Webb was supported by a Sigma-Aldrich sponsored scholarship during the time that the presented work was carried out at UWM. DOZN™ is an online green chemistry tool developed by Sigma-Aldrich.

## Supplementary Material

SU-001-D3SU00005B-s001
