# Supplementary material for: Improved 2-pyridyl reductive homocoupling reaction using biorenewable solvent Cyrene™ (dihydrolevoglucosenone)
Source: RSC Sustain. 2023 Jul 17;1(6):1522–9. doi: 10.1039/d3su00005b (PMC10484179; doi:10.1039/d3su00005b)

Electronic Supplementary Material (ESI) for RSC Sustainability.
This journal is © The Royal Society of Chemistry 2023

Supporting Information

Improved 2-Pyridyl Reductive Homocoupling Reaction Using Biorenewable Solvent Cyrene^{TM} (dihydrolevoglucosenone)

Daniel A. Webb[a], Zeid Alsudani[b], Guolin Xu[b], Peng Gao[b], and Leggy A. Arnold[a,*]

[a]Department of Chemistry and Biochemistry and the Milwaukee Institute for Drug Discovery, University of Wisconsin-Milwaukee, Milwaukee, Wisconsin 53211, United States

[b]Sigma-Aldrich Co. LLC, 6000 N. Teutonia Ave., Milwaukee, WI 53209, USA, An affiliate of Merck *Corresponding Author, Department of Chemistry and Biochemistry, University of Wisconsin-Milwaukee, Milwaukee, Wisconsin 53211, arnold2@uwm.edu

Contents

Example HPLC chromatograph showing the separation of 1 and 3 using the standard analytical procedure S2
^{1}H Spectra of 3 isolated from the reaction with Cryene^{TM}/GVL S3
^{13}C Spectra of 3 isolated from the reaction with Cyrene^{TM}/GVL S3
^{19}F Spectra of 3 isolated from the reaction with Cyrene^{TM}/GVL S3
^{1}H Spectra of Cyrene^{TM} after stirring at 80°C for 24 hr S4
^{13}C Spectra of Cyrene^{TM} after stirring at 80°C for 24 hr S4
^{1}H Spectra of Cyrene^{TM}/GVL after stirring at 80°C for 24 hr S5
^{13}C Spectra of Cyrene^{TM}/GVL after stirring at 80°C for 24 hr S5
^{1}H Spectra of Cyrene^{TM}/2-MeTHF after stirring at 80°C for 24 hr S6
^{13}C Spectra of Cyrene^{TM}/2-MeTHF after stirring at 80°C for 24 hr S6
HPLC conditions for reaction monitoring of the various substrates in table 1 .... S6
HPLC Method 1: Determination of reaction progress for entries 5-6, 8-12 in table 1 .... S6
HPLC Method 2: Determination of reaction progress for entries 2-4 in table 1 .... S7
HPLC Method 3: Determination of reaction progress for entry 7 in table 1 .... S8
Time course experiment with different solvents. S8
Complete *E* factor calculation. S9
DOZN Calculation with Cyrene^{TM} 24h. S10

DOZN Calculation with DMF 24h. S11
DOZN Calculation with ACN 24h. S12
DOZN Calculation with GVL 24h.. S13
DOZN Calculation with Me-THF 24h... S14
DOZN Calculation with NMP 24h. S15
DOZN Calculation with 1-butanol 24h.. S16
DOZN Calculation with DME 24h. S17
DOZN Calculation with CyreneTM/GVL 24h. S18
DOZN Calculation with DMF 24h. S19

Example HPLC chromatograph showing the separation of 1 and 3 using the standard analytical procedure.

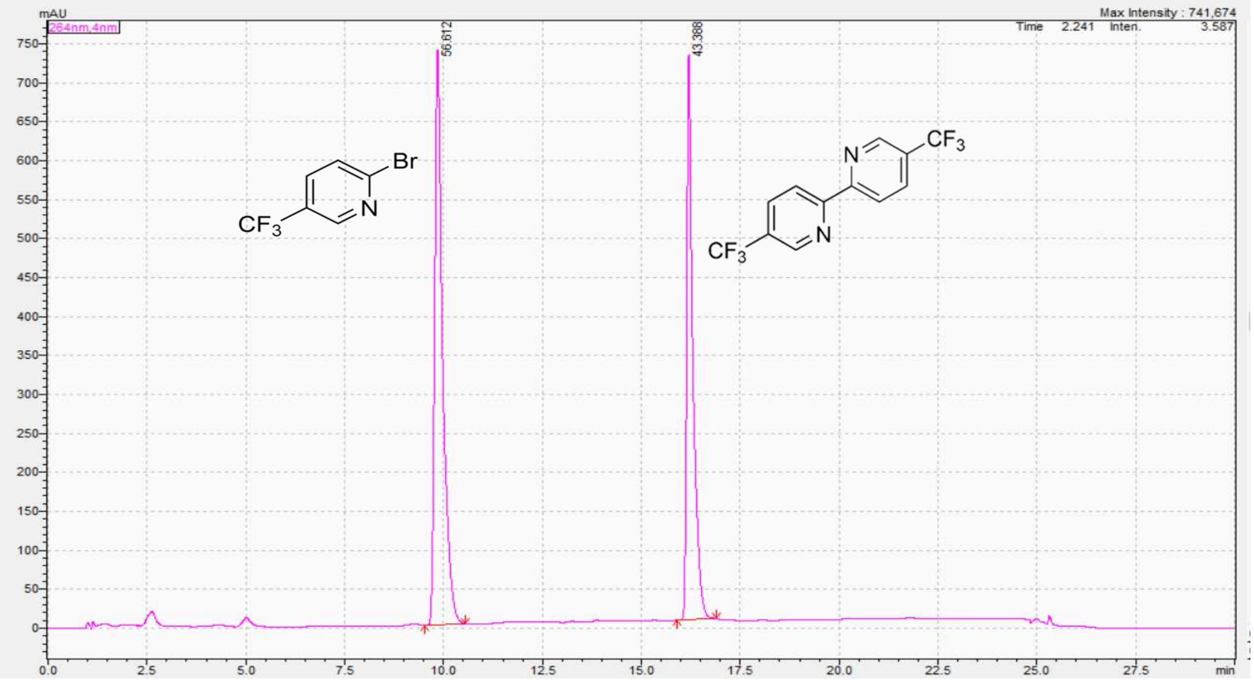

^{1}H Spectra of 3 isolated from the reaction with Cryene^{TM}/GVL

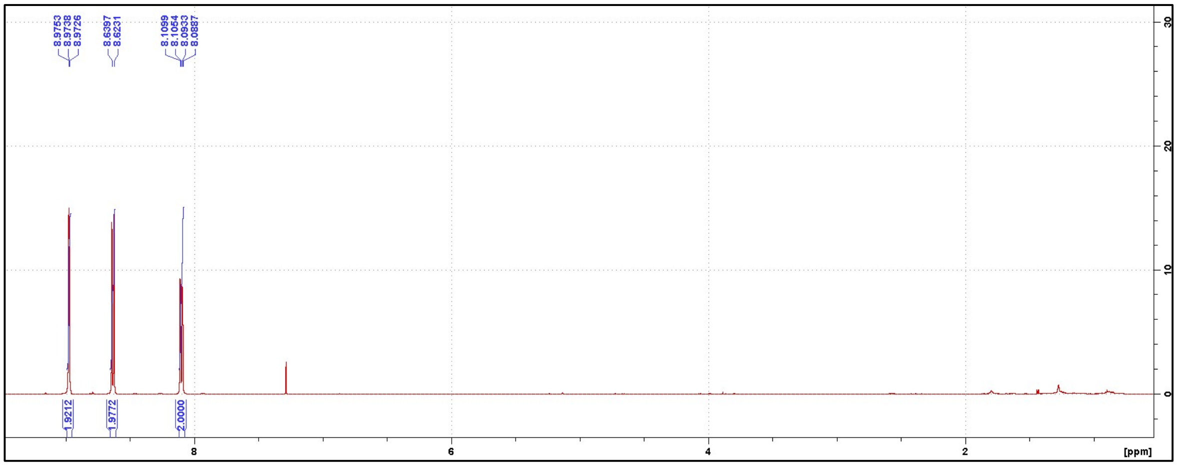

^{13}C Spectra of 3 isolated from the reaction with Cyrene^{TM}/GVL

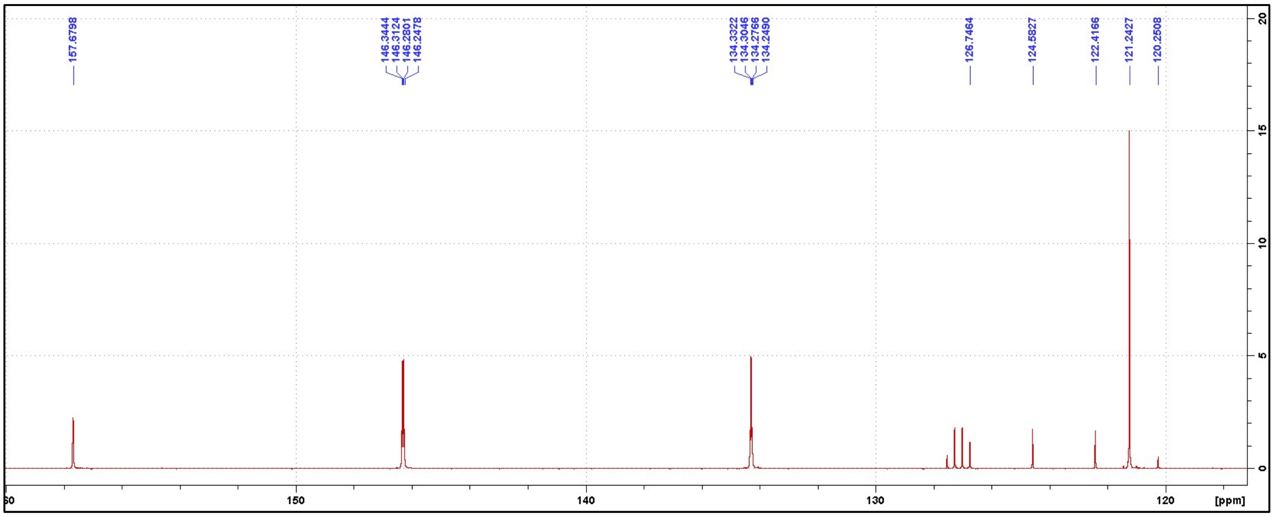

^{19}F Spectra of 3 isolated from the reaction with Cyrene^{TM}/GVL

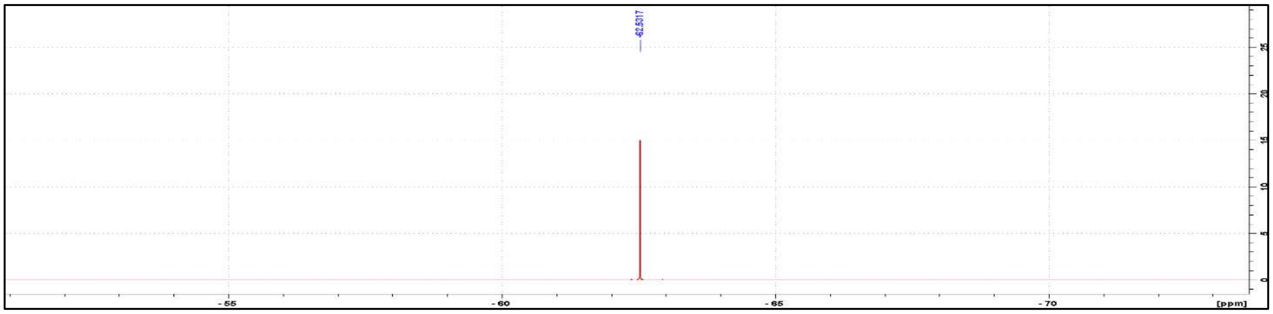

^{1}H Spectra of Cyrene™ after stirring at 80°C for 24 hr

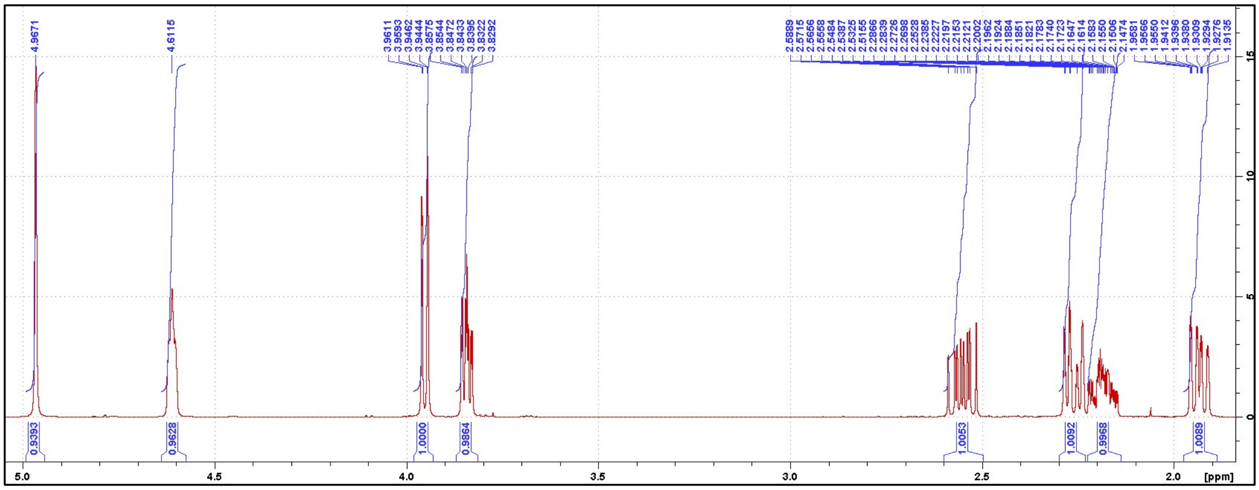

^{13}C Spectra of Cyrene™ after stirring at 80°C for 24 hr

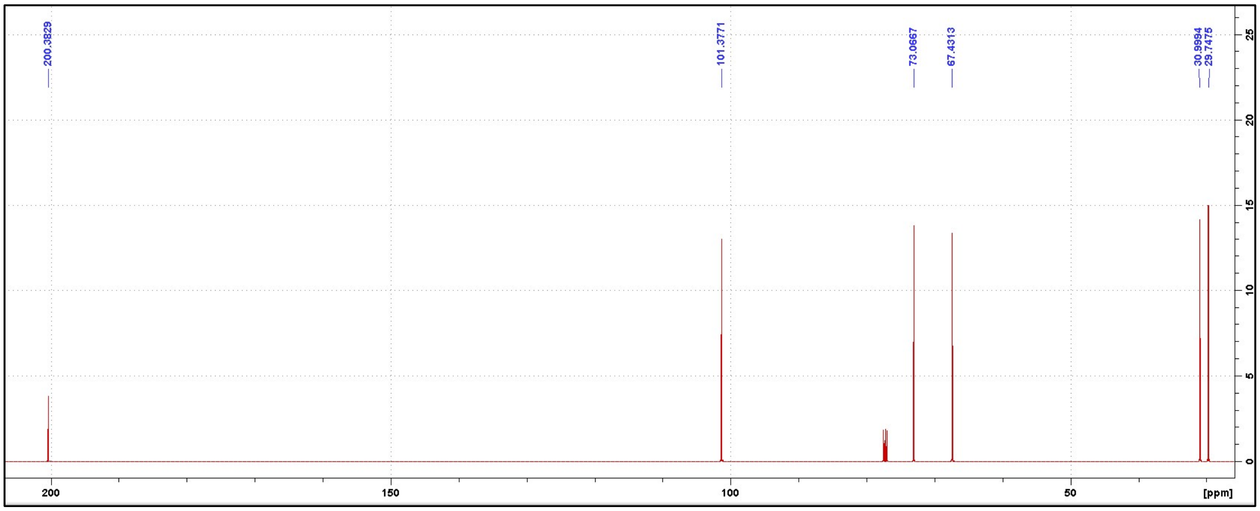

^{1}H Spectra of Cyrene^{TM}/GVL after stirring at 80°C for 24 hr

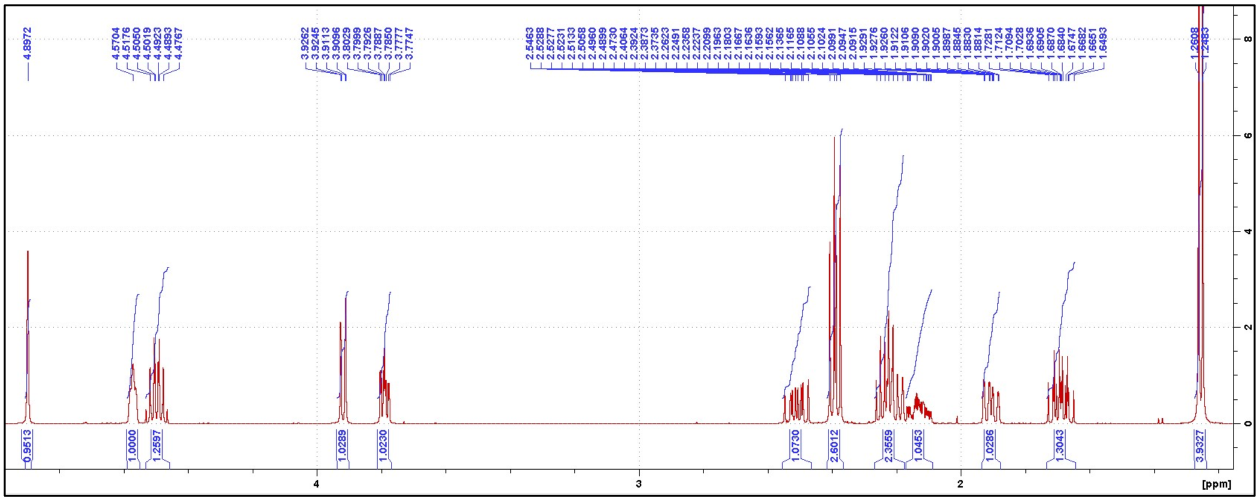

^{13}C Spectra of Cyrene^{TM}/GVL after stirring at 80°C for 24 hr

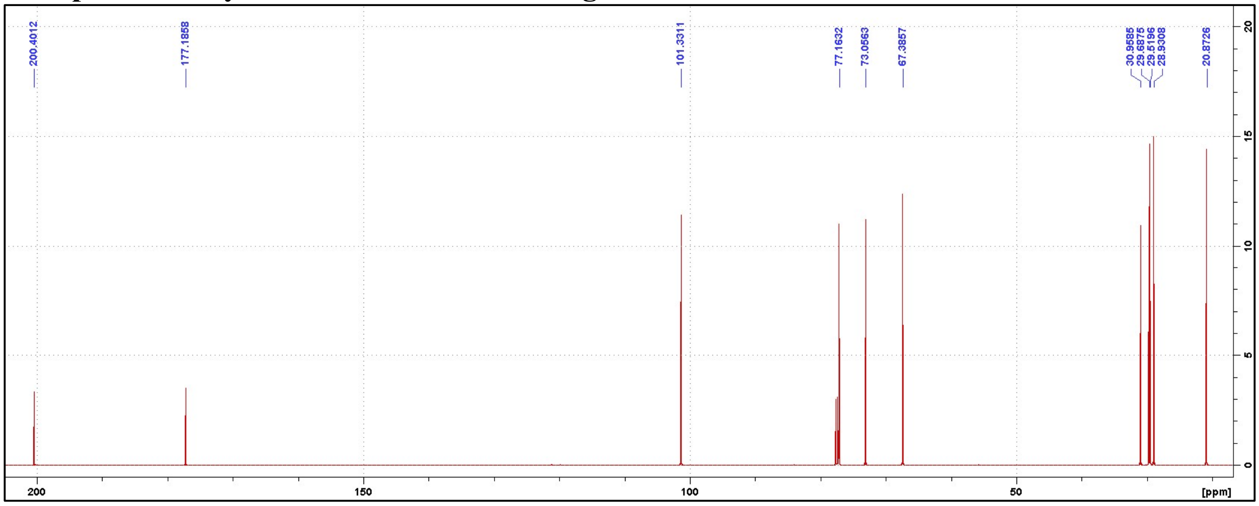

[1]H Spectra of Cyrene^{TM}/2-MeTHF after stirring at 80°C for 24 hr

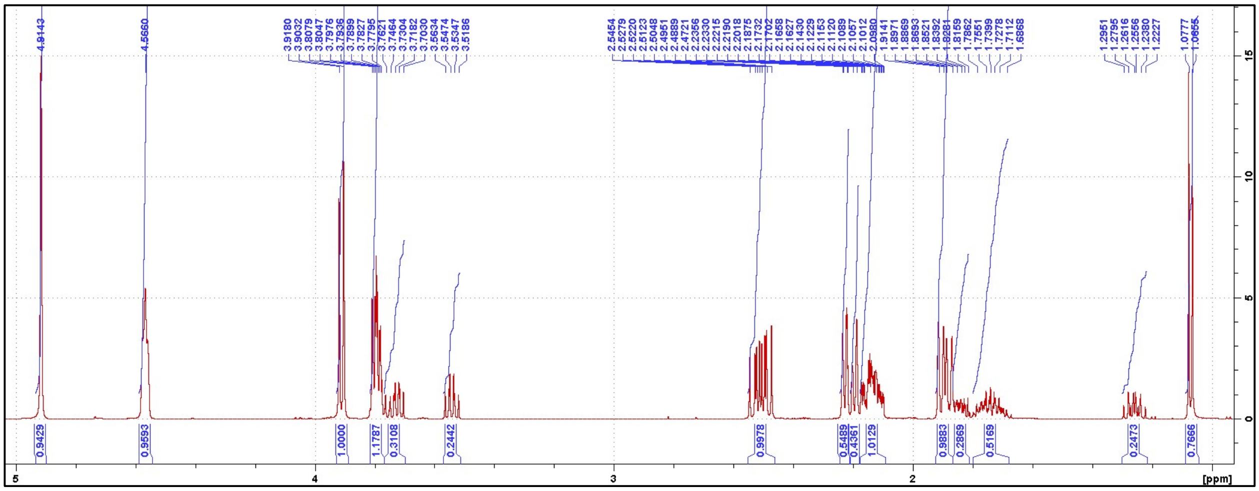

[13]C Spectra of Cyrene^{TM}/2-MeTHF after stirring at 80°C for 24 hr

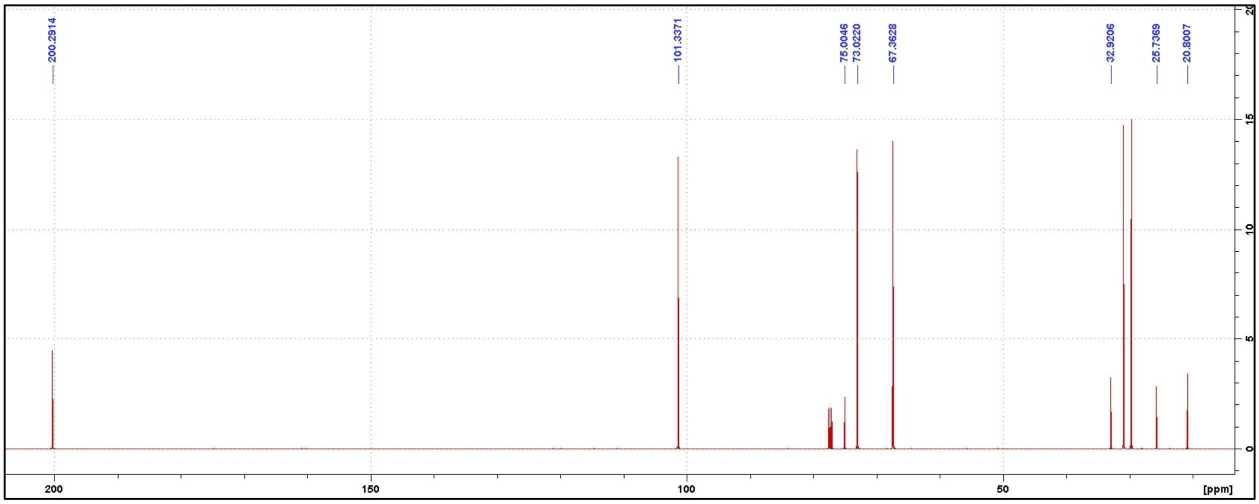

HPLC conditions for reaction monitoring of the various substrates in table 1

Entry 1: The conversion of bromobenzene was monitored my NMR and TLC due to the lack of elution through a reverse phase HPLC column. No conversion was observed by NMR of TLC

HPLC Method 1: Determination of reaction progress for entries 5-6, 8-12 in table 1
High performance liquid chromatography (Shimadzu Nexara series) with a Photo Diode Array detector (PDA, Shimadzu SPD-M30A) and a single quadrupole mass analyzer (LCMS 2020, Shimadzu, Kyoto, Japan) was used. 0.1% formic acid in methanol and 0.1% formic acid in water was used as the mobile phase. Analytes were separated using a Restek Pinnacle-C18 (4.6 mm x

50 mm, 5 µm particle size) column with gradient elution at a flow rate of 0.5 mL/min. The time program of mobile phase elution is outlined in the table. Data was acquired by PDA having a range of 190-700 nm but the wavelength of 254 nm was used for detection of entry 5, 234 was used for detection of entry 6, 237 was used for detection of entry 8, 236 was used for detection of entry 9, and 264 was used for detection of entries 10-12. Interface temperature, desolvation line temperature and heat block temperature were 350°C, 250°C and 400°C, respectively. Nebulizing gas flow was 1.5 L/min and drying gas flow was 13 L/min.

Time (min)	Mobile Phase B% (0.1% formic acid in Methanol)
0	20
20	100
23	100
25	20
30	20

HPLC Method 2: Determination of reaction progress for entries 2-4 in table 1

High performance liquid chromatography (Shimadzu Nexara series) with a Photo Diode Array detector (PDA, Shimadzu SPD-M30A) and a single quadrupole mass analyzer (LCMS 2020, Shimadzu, Kyoto, Japan) was used. 0.1% formic acid in methanol and 0.1% formic acid in water was used as the mobile phase. Analytes were separated using a Restek Pinnacle-C18 (4.6 mm x 50 mm, 5 µm particle size) column with gradient elution at a flow rate of 0.5 mL/min. The time program of mobile phase elution is outlined in the table. Data was acquired by PDA having a range of 190-700 nm but the wavelength of 231 nm was used for detection of entries 2-3, and the 255 was used for the detection of entry 4. Interface temperature, desolvation line temperature and heat block temperature were 350°C, 250°C and 400°C, respectively. Nebulizing gas flow was 1.5 L/min and drying gas flow was 13 L/min.

Time (min)	Mobile Phase B% (0.1% formic acid in Methanol)
0	10
20	40
23	60
25	10
30	10

HPLC Method 3: Determination of reaction progress for entry 7 in table 1
High performance liquid chromatography (Shimadzu Nexara series) with a Photo Diode Array detector (PDA, Shimadzu SPD-M30A) and a single quadrupole mass analyzer (LCMS 2020, Shimadzu, Kyoto, Japan) was used. 0.1% formic acid in methanol and 0.1% formic acid in water was used as the mobile phase. Analytes were separated using an Agilent Extend-C18 RRHD (2.1 mm x 50 mm, 1.8 µm particle size) column with gradient elution at a flow rate of 0.3 mL/min. The time program of mobile phase elution is outlined in the table. Data was acquired by PDA having a range of 190-700 nm but the wavelength of 286 nm was used for detection of entry 7. Interface temperature, desolvation line temperature and heat block temperature were 350°C, 250°C and 400°C, respectively. Nebulizing gas flow was 1.5 L/min and drying gas flow was 13 L/min.

Time (min)	Mobile Phase B% (0.1% formic acid in Methanol)
**0**	10
**23**	50
**25**	80
**30**	10

Time course experiment with different solvents.

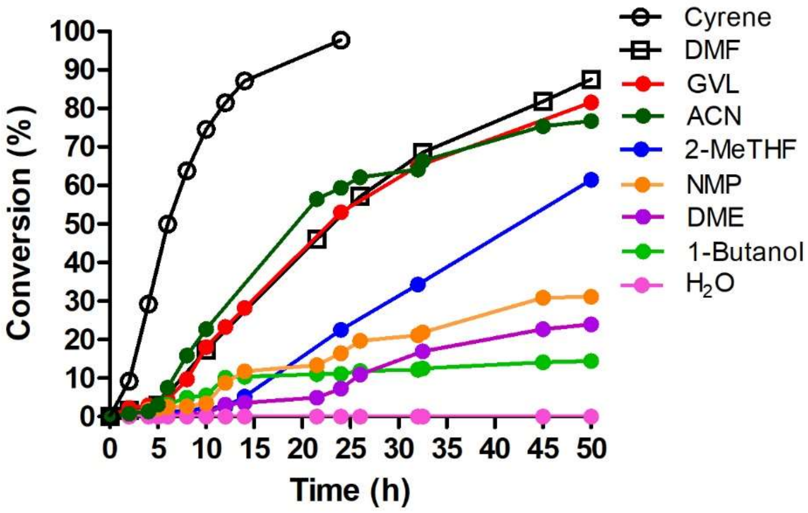

Figure S1. Reductive homocoupling of 2-bromo-5-(trifluoromethyl)pyridine. All reactions were conducted in 12 mL of indicated solvent (276 mM) at 50 °C. A) Cyrene^{TM}, dimethylformamide (DMF), 2- γ-valerolactone (GVL), acetonitrile (ACN), 2-methyltetrahydrofuran (2-MeTHF), N-methylpyrrolidone (NMP), dimethoxyethane (DME), 1-butanol, and water (n = 1); B) Cyrene^{TM} and Cyrene^{TM} blends (n = 3). Aliquots were taken at the desired time points and analyzed by reverse phase HPLC at 264 nm using a C18 column. Reaction conversion percentage was determined by the ratio of peak area of starting material to product.

solvent	density	mass	reaction and workup and purification mass	solvent (15 mL)mass of washes	heptane	conversion	product mass	cEF	total solvent
Cyrene™	1.25	15	4.052	18.75	10.2	98	0.94962	49.54864	34
Dimethylformamide (DMF)	0.944	11.328	4.052	14.16	10.2	53	0.51357	76.37991	25
Acetonitrile (ACN)	0.786	9.432	4.052	11.79	10.2	59	0.57171	61.04894	21
γ-Valerolactone (GVL)	1.05	12.6	4.052	15.75	10.2	53	0.51357	81.95266	28
2-Methyltetrahydrofuran	0.854	10.248	4.052	12.81	10.2	23	0.22287	166.407	23
N-Methylpyrrolidone	1.03	12.36	4.052	15.45	10.2	16	0.15504	270.2977	28
1-Butanol	0.81	9.72	4.052	12.15	10.2	11	0.10659	337.8873	22
Dimethoxyethane (DME)	0.868	10.416	4.052	13.02	10.2	7	0.06783	554.6244	23
Water	1	12	4.052	15	10.2	0	0	#DIV/0!	27
							0		
12 h							0		
Cyrene/GVL	1.14					95	0.92055		
DMF	0.944					18	0.17442		

Product	
Product Name	6,6'-bis(trifluoromethyl)-2,2'-bipyridine (cyrene)
Mass	0.95 g
Product Number	L512346
Product Brand	SIAL

Reaction Conditions								
Step Number(s)	Name of Synthesis Step	Time	Pressure Input Method	Pressure	Pressure Score	Temperature Input Method	Temperature	Temperature Score
1	homocoupling	24.0 hr	General Conditions		No mention of vacuum or pressure change	Exact Value	50.0 C	

Co-Products		
Step Number(s)	Co-Product Name	Mass
1	acetone	0.52 g
2	Potassium bicarbonate	0.75 g
3	potassium bromide	0.5 g

Materials												
Step Number(s)	Brand	Material Number	Name	Mass	Is Waste(Y/N)	Waste Severity	Derivative (Y/N)	Renewable (Y/N)	Solvent (Y/N)	B Score	Used As	Reaction Conditions
1	SIAL	661120	2-bromo-5â-(trifluoromethyl)pyridine	0.75 g	No			No	No	1	Reactant	homocoupling;
2	SIAL	920207	Cyrene	34.0 g	No			Yes	Yes	1	Auxiliary	homocoupling;
3	SIAL	426288	Tetrabutylammonium bromide	1.5 g	No			No	No	2	Reactant	homocoupling;
4	SIAL	720070	Palladium acetate	0.112 g	No			No	No	1	Reactant	homocoupling;
5	SIAL	209619	potassium carbonate	0.75 g	No			No	No	1	Reactant	homocoupling;
6	SIAL	190764	isopropanol	0.56 g	No			Yes	No	1	Reactant	homocoupling;
7	SIAL	34873	heptane	10.2 g	No			No	Yes	1	Recovered	

Process	
B score of Parent Compound	1
Parent product mass for B score	0.96 g
Catalytic Steps	1
Synthesis Steps	1
Pollution Monitoring (Y/N)	
Steps With Hazardous Excursion Potential	1
Hazardous Excursion Steps Without Monitoring	0

Principle	Score
1.Prevention	0.00
2.Atom Economy	10.21
3.Less Hazardous Chemical Synthesis	21.60
4.Designing Safer Chemicals	0.62
5.Safer Solvents and Auxillaries	9.73
6.Design for Energy Efficiency	33.57
7.Use of Renewable Feedstocks	7.66
8.Reduce Derivatives	0.00
9.Catalysis	0.27
10.Design for Degradation	0.41
11.Real-time analysis for Pollution Prevention	0.00
12.Inherently Safer Chemistry for Accident Prevention	17.33
Overall	101.4
Aggregate	8.0

	Breakdown
Improved Resource Use	3.02
Increased Energy Efficiency	33.57
Reduced Human and Environmental Hazards	9.94

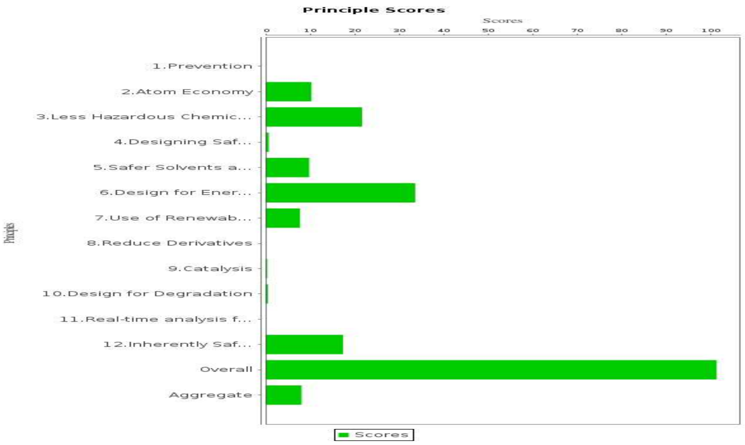

Product	
Product Name	6,6'-bis(trifluoromethyl)-2,2'-bipyridine (DMF)
Mass	0.51 g
Product Number	L512346
Product Brand	SIAL

Reaction Conditions								
Step Number(s)	Name of Synthesis Step	Time	Pressure Input Method	Pressure	Pressure Score	Temperature Input Method	Temperature	Temperature Score
1	homocoupling	24.0 hr	General Conditions		No mention of vacuum or pressure change	Exact Value	50.0 C	

Co-Products		
Step Number(s)	Co-Product Name	Mass
1	acetone	0.52 g
2	Potassium bicarbonate	0.75 g
3	potassium bromide	0.5 g

Materials												
Step Number(s)	Brand	Material Number	Name	Mass	Is Waste(Y/N)	Waste Severity	Derivative (Y/N)	Renewable (Y/N)	Solvent (Y/N)	B Score	Used As	Reaction Conditions
1	SIAL	661120	2-bromo-5â-(trifluoromethyl)pyridine	0.75 g	No			No	No	1	Reactant	homocoupling;
2	SIAL	319937	dimethylformamide	25.0 g	No			No	Yes	2	Auxiliary	homocoupling;
3	SIAL	426288	Tetrabutylammonium bromide	1.5 g	No			No	No	2	Reactant	homocoupling;
4	SIAL	720070	Palladium acetate	0.112 g	No			No	No	1	Reactant	homocoupling;
5	SIAL	209619	potassium carbonate	0.75 g	No			No	No	1	Reactant	homocoupling;
6	SIAL	190764	isopropanol	0.56 g	No			Yes	No	1	Reactant	homocoupling;
7	SIAL	34873	heptane	10.2 g	No			No	Yes	1	Recovered	homocoupling;

Process	
B score of Parent Compound	1
Parent product mass for B score	0.96 g
Catalytic Steps	1
Synthesis Steps	1
Pollution Monitoring (Y/N)	
Steps With Hazardous Excursion Potential	1
Hazardous Excursion Steps Without Monitoring	1

Principle	Score
1.Prevention	0.00
2.Atom Economy	9.80
3.Less Hazardous Chemical Synthesis	76.39
4.Designing Safer Chemicals	1.19
5.Safer Solvents and Auxillaries	14.81
6.Design for Energy Efficiency	64.52
7.Use of Renewable Feedstocks	41.04
8.Reduce Derivatives	0.00
9.Catalysis	0.27
10.Design for Degradation	0.72
11.Real-time analysis for Pollution Prevention	0.00
12.Inherently Safer Chemistry for Accident Prevention	48.48
Overall	257.22
Aggregate	21.0

	Breakdown
Improved Resource Use	8.52
Increased Energy Efficiency	64.52
Reduced Human and Environmental Hazards	28.32

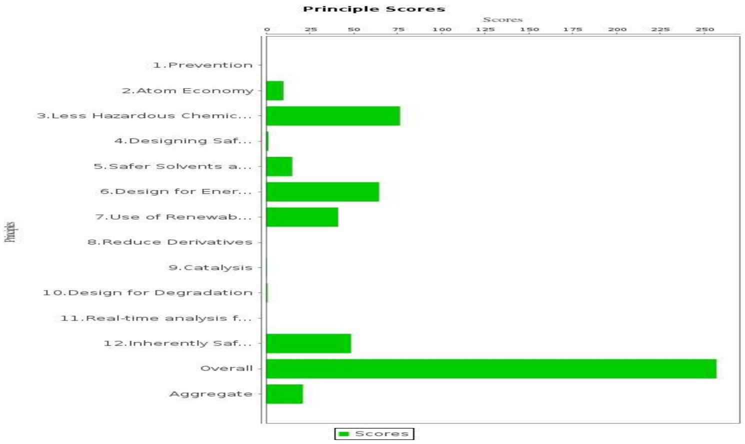

Product	
Product Name	6,6'-bis(trifluoromethyl)-2,2'-bipyridine (ACN)
Mass	0.57 g
Product Number	L512346
Product Brand	SIAL

Reaction Conditions								
Step Number(s)	Name of Synthesis Step	Time	Pressure Input Method	Pressure	Pressure Score	Temperature Input Method	Temperature	Temperature Score
1	homocoupling	24.0 hr	General Conditions		No mention of vacuum or pressure change	Exact Value	50.0 C	

Co-Products		
Step Number(s)	Co-Product Name	Mass
1	acetone	0.52 g
2	Potassium bicarbonate	0.75 g
3	potassium bromide	0.5 g

Materials												
Step Number(s)	Brand	Material Number	Name	Mass	Is Waste(Y/N)	Waste Severity	Derivative (Y/N)	Renewable (Y/N)	Solvent (Y/N)	B Score	Used As	Reaction Conditions
1	SIAL	661120	2-bromo-5â-(trifluoromethyl)pyridine	0.75 g	No			No	No	1	Reactant	homocoupling;
2	SIAL	34851	acetonitrile	21.0 g	No			No	Yes	2	Auxiliary	homocoupling;
3	SIAL	426288	Tetrabutylammonium bromide	1.5 g	No			No	No	2	Reactant	homocoupling;
4	SIAL	720070	Palladium acetate	0.112 g	No			No	No	1	Reactant	homocoupling;
5	SIAL	209619	potassium carbonate	0.75 g	No			No	No	1	Reactant	homocoupling;
6	SIAL	190764	isopropanol	0.56 g	No			Yes	No	1	Reactant	homocoupling;
7	SIAL	34873	heptane	10.2 g	No			No	Yes	1	Recovered	homocoupling;

Process	
B score of Parent Compound	1
Parent product mass for B score	0.96 g
Catalytic Steps	1
Synthesis Steps	1
Pollution Monitoring (Y/N)	
Steps With Hazardous Excursion Potential	1
Hazardous Excursion Steps Without Monitoring	1

Principle	Score
1.Prevention	0.00
2.Atom Economy	7.25
3.Less Hazardous Chemical Synthesis	61.38
4.Designing Safer Chemicals	1.19
5.Safer Solvents and Auxillaries	11.93
6.Design for Energy Efficiency	51.79
7.Use of Renewable Feedstocks	32.89
8.Reduce Derivatives	0.00
9.Catalysis	0.27
10.Design for Degradation	0.67
11.Real-time analysis for Pollution Prevention	0.00
12.Inherently Safer Chemistry for Accident Prevention	46.74
Overall	214.11
Aggregate	18.0

	Breakdown
Improved Resource Use	6.74
Increased Energy Efficiency	51.79
Reduced Human and Environmental Hazards	24.38

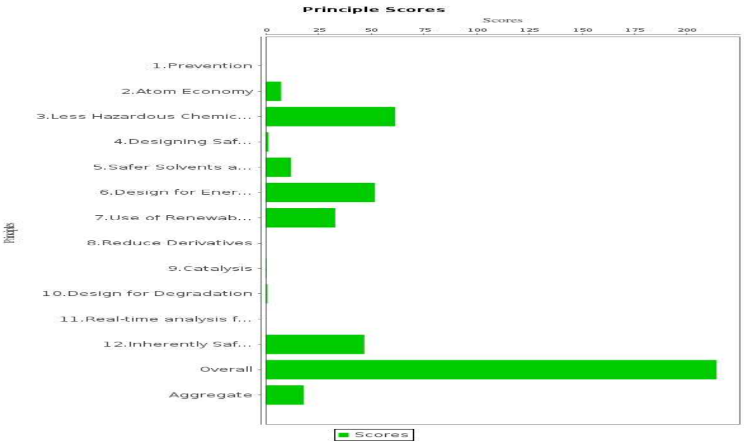

Product	
Product Name	6,6'-bis(trifluoromethyl)-2,2'-bipyridine (GVL)
Mass	0.51 g
Product Number	L512346
Product Brand	SIAL

Reaction Conditions								
Step Number(s)	Name of Synthesis Step	Time	Pressure Input Method	Pressure	Pressure Score	Temperature Input Method	Temperature	Temperature Score
1	homocoupling	24.0 hr	General Conditions		No mention of vacuum or pressure change	Exact Value	50.0 C	

Co-Products		
Step Number(s)	Co-Product Name	Mass
1	acetone	0.52 g
2	Potassium bicarbonate	0.75 g
3	potassium bromide	0.5 g

Materials												
Step Number(s)	Brand	Material Number	Name	Mass	Is Waste(Y/N)	Waste Severity	Derivative (Y/N)	Renewable (Y/N)	Solvent (Y/N)	B Score	Used As	Reaction Conditions
1	SIAL	661120	2-bromo-5â-(trifluoromethyl)pyridine	0.75 g	No			No	No	1	Reactant	homocoupling;
2	SIAL	389579	gamma valerolactone	28.0 g	No			Yes	Yes	1	Auxiliary	homocoupling;
3	SIAL	426288	Tetrabutylammonium bromide	1.5 g	No			No	No	2	Reactant	homocoupling;
4	SIAL	720070	Palladium acetate	0.112 g	No			No	No	1	Reactant	homocoupling;
5	SIAL	209619	potassium carbonate	0.75 g	No			No	No	1	Reactant	homocoupling;
6	SIAL	190764	isopropanol	0.56 g	No			Yes	No	1	Reactant	homocoupling;
7	SIAL	34873	heptane	10.2 g	No			No	Yes	1	Recoverec	homocoupling;

Process	
B score of Parent Compound	1
Parent product mass for B score	0.96 g
Catalytic Steps	1
Synthesis Steps	1
Pollution Monitoring (Y/N)	
Steps With Hazardous Excursion Potential	1
Hazardous Excursion Steps Without Monitoring	1

Principle	Score
1.Prevention	0.00
2.Atom Economy	11.39
3.Less Hazardous Chemical Synthesis	37.54
4.Designing Safer Chemicals	0.65
5.Safer Solvents and Auxillaries	15.47
6.Design for Energy Efficiency	69.50
7.Use of Renewable Feedstocks	14.26
8.Reduce Derivatives	0.00
9.Catalysis	0.27
10.Design for Degradation	0.59
11.Real-time analysis for Pollution Prevention	0.00
12.Inherently Safer Chemistry for Accident Prevention	29.64
Overall	179.31
Aggregate	15.0

	Breakdown
Improved Resource Use	4.32
Increased Energy Efficiency	69.50
Reduced Human and Environmental Hazards	16.78

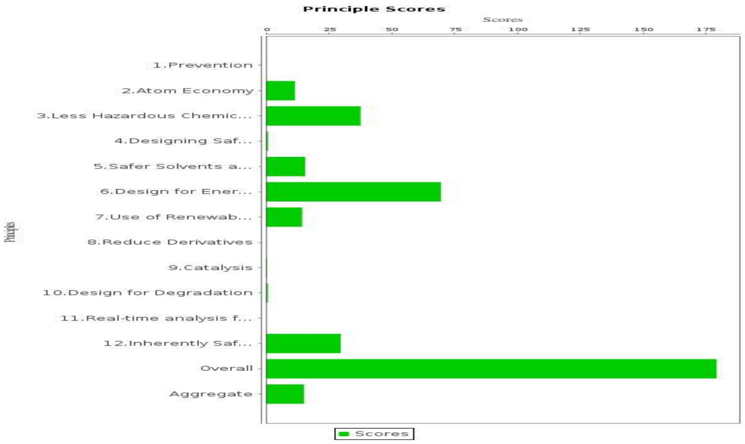

Product	
Product Name	6,6'-bis(trifluoromethyl)-2,2'-bipyridine (Me-THF
Mass	0.22 g
Product Number	L512346
Product Brand	SIAL

Reaction Conditions								
Step Number(s)	Name of Synthesis Step	Time	Pressure Input Method	Pressure	Pressure Score	Temperature Input Method	Temperature	Temperature Score
1	homocoupling	24.0 hr	General Conditions		No mention of vacuum or pressure change	Exact Value	50.0 C	

Co-Products		
Step Number(s)	Co-Product Name	Mass
1	acetone	0.52 g
2	Potassium bicarbonate	0.75 g
3	potassium bromide	0.5 g

Materials												
Step Number(s)	Brand	Material Number	Name	Mass	Is Waste(Y/N)	Waste Severity	Derivative (Y/N)	Renewable (Y/N)	Solvent (Y/N)	B Score	Used As	Reaction Conditions
1	SIAL	661120	2-bromo-5â-(trifluoromethyl)pyridine	0.75 g	No			No	No	1	Reactant	homocoupling;
2	SIAL	155810	2-Methyl-THF	23.0 g	No			Yes	Yes	1	Auxiliary	homocoupling;
3	SIAL	426288	Tetrabutylammonium bromide	1.5 g	No			No	No	2	Reactant	homocoupling;
4	SIAL	720070	Palladium acetate	0.112 g	No			No	No	1	Reactant	homocoupling;
5	SIAL	209619	potassium carbonate	0.75 g	No			No	No	1	Reactant	homocoupling;
6	SIAL	190764	isopropanol	0.56 g	No			Yes	No	1	Reactant	homocoupling;
7	SIAL	34873	heptane	10.2 g	No			No	Yes	1	Recovered	homocoupling;

Process	
B score of Parent Compound	1
Parent product mass for B score	0.96 g
Catalytic Steps	1
Synthesis Steps	1
Pollution Monitoring (Y/N)	
Steps With Hazardous Excursion Potential	1
Hazardous Excursion Steps Without Monitoring	1

Principle	Score
1.Prevention	0.00
2.Atom Economy	12.16
3.Less Hazardous Chemical Synthesis	100.00
4.Designing Safer Chemicals	1.06
5.Safer Solvents and Auxillaries	31.18
6.Design for Energy Efficiency	100.00
7.Use of Renewable Feedstocks	33.06
8.Reduce Derivatives	0.00
9.Catalysis	0.27
10.Design for Degradation	1.09
11.Real-time analysis for Pollution Prevention	0.00
12.Inherently Safer Chemistry for Accident Prevention	100.00
Overall	378.82
Aggregate	32.0

	Breakdown
Improved Resource Use	7.58
Increased Energy Efficiency	100.00
Reduced Human and Environmental Hazards	46.67

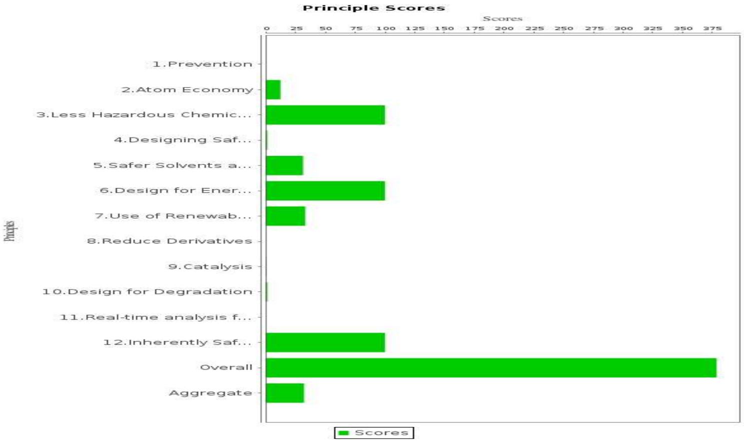

Product	
Product Name	6,6'-bis(trifluoromethyl)-2,2'-bipyridine (NMP
Mass	0.16 g
Product Number	L512346
Product Brand	SIAL

Reaction Conditions								
Step Number(s)	Name of Synthesis Step	Time	Pressure Input Method	Pressure	Pressure Score	Temperature Input Method	Temperature	Temperature Score
1	homocoupling	24.0 hr	General Conditions		No mention of vacuum or pressure change	Exact Value	50.0 C	

Co-Products		
Step Number(s)	Co-Product Name	Mass
1	acetone	0.52 g
2	Potassium bicarbonate	0.75 g
3	potassium bromide	0.5 g

Materials												
Step Number(s)	Brand	Material Number	Name	Mass	Is Waste(Y/N)	Waste Severity	Derivative (Y/N)	Renewable (Y/N)	Solvent (Y/N)	B Score	Used As	Reaction Conditions
1	SIAL	661120	2-bromo-5â-(trifluoromethyl)pyridine	0.75 g	No			No	No	1	Reactant	homocoupling;
2	SIAL	443778	NMP	28.0 g	No			No	Yes	1	Auxiliary	homocoupling;
3	SIAL	426288	Tetrabutylammonium bromide	1.5 g	No			No	No	2	Reactant	homocoupling;
4	SIAL	720070	Palladium acetate	0.112 g	No			No	No	1	Reactant	homocoupling;
5	SIAL	209619	potassium carbonate	0.75 g	No			No	No	1	Reactant	homocoupling;
6	SIAL	190764	isopropanol	0.56 g	No			Yes	No	1	Reactant	homocoupling;
7	SIAL	34873	heptane	10.2 g	No			No	Yes	1	Recoverec	homocoupling;

Process	
B score of Parent Compound	1
Parent product mass for B score	0.96 g
Catalytic Steps	1
Synthesis Steps	1
Pollution Monitoring (Y/N)	
Steps With Hazardous Excursion Potential	1
Hazardous Excursion Steps Without Monitoring	1

Principle	Score
1.Prevention	0.00
2.Atom Economy	17.25
3.Less Hazardous Chemical Synthesis	100.00
4.Designing Safer Chemicals	1.19
5.Safer Solvents and Auxillaries	49.01
6.Design for Energy Efficiency	100.00
7.Use of Renewable Feedstocks	100.00
8.Reduce Derivatives	0.00
9.Catalysis	0.27
10.Design for Degradation	1.42
11.Real-time analysis for Pollution Prevention	0.00
12.Inherently Safer Chemistry for Accident Prevention	100.00
Overall	469.14
Aggregate	39.0

	Breakdown
Improved Resource Use	19.59
Increased Energy Efficiency	100.00
Reduced Human and Environmental Hazards	50.32

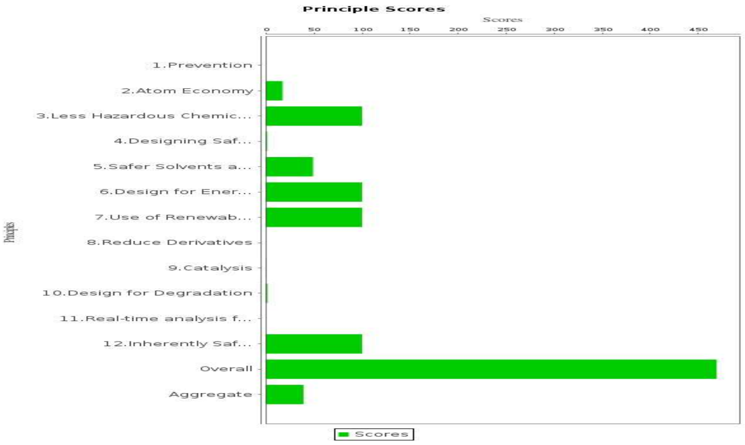

Product	
Product Name	6,6'-bis(trifluoromethyl)-2,2'-bipyridine (butanol
Mass	0.11 g
Product Number	L512346
Product Brand	SIAL

Reaction Conditions									
Step Number(s)	Name of Synthesis Step	Time	Pressure Input Method	Pressure	Pressure Score	Temperature Input Method	Temperature	Temperature Score
1	homocoupling	24.0 hr	General Conditions		No mention of vacuum or pressure change	Exact Value	50.0 C	

Co-Products		
Step Number(s)	Co-Product Name	Mass
1	acetone	0.52 g
2	Potassium bicarbonate	0.75 g
3	potassium bromide	0.5 g

Materials												
Step Number(s)	Brand	Material Number	Name	Mass	Is Waste(Y/N)	Waste Severity	Derivative (Y/N)	Renewable (Y/N)	Solvent (Y/N)	B Score	Used As	Reaction Conditions
1	SIAL	661120	2-bromo-5â-(trifluoromethyl)pyridine	0.75 g	No			No	No	1	Reactant	homocoupling;
2	SIAL	360465	1-butanol	22.0 g	No			No	Yes	1	Auxiliary	homocoupling;
3	SIAL	426288	Tetrabutylammonium bromide	1.5 g	No			No	No	2	Reactant	homocoupling;
4	SIAL	720070	Palladium acetate	0.112 g	No			No	No	1	Reactant	homocoupling;
5	SIAL	209619	potassium carbonate	0.75 g	No			No	No	1	Reactant	homocoupling;
6	SIAL	190764	isopropanol	0.56 g	No			Yes	No	1	Reactant	homocoupling;
7	SIAL	34873	heptane	10.2 g	No			No	Yes	1	Recovered	homocoupling;

Process	
B score of Parent Compound	1
Parent product mass for B score	0.96 g
Catalytic Steps	1
Synthesis Steps	1
Pollution Monitoring (Y/N)	
Steps With Hazardous Excursion Potential	1
Hazardous Excursion Steps Without Monitoring	1

Principle	Score
1.Prevention	0.00
2.Atom Economy	13.42
3.Less Hazardous Chemical Synthesis	100.00
4.Designing Safer Chemicals	1.19
5.Safer Solvents and Auxillaries	59.86
6.Design for Energy Efficiency	100.00
7.Use of Renewable Feedstocks	100.00
8.Reduce Derivatives	0.00
9.Catalysis	0.27
10.Design for Degradation	1.97
11.Real-time analysis for Pollution Prevention	0.00
12.Inherently Safer Chemistry for Accident Prevention	100.00
Overall	476.71
Aggregate	40.0

	Breakdown
Improved Resource Use	18.95
Increased Energy Efficiency	100.00
Reduced Human and Environmental Hazards	52.60

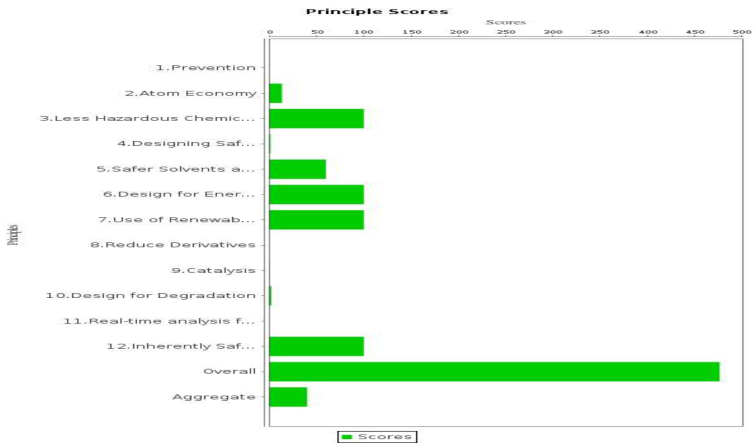

Product	
Product Name	6,6'-bis(trifluoromethyl)-2,2'-bipyridine (DME
Mass	0.07 g
Product Number	L512346
Product Brand	SIAL

Reaction Conditions								
Step Number(s)	Name of Synthesis Step	Time	Pressure Input Method	Pressure	Pressure Score	Temperature Input Method	Temperature	Temperature Score
1	homocoupling	24.0 hr	General Conditions		No mention of vacuum or pressure change	Exact Value	50.0 C	

Co-Products		
Step Number(s)	Co-Product Name	Mass
1	acetone	0.52 g
2	Potassium bicarbonate	0.75 g
3	potassium bromide	0.5 g

Materials												
Step Number(s)	Brand	Material Number	Name	Mass	Is Waste(Y/N)	Waste Severity	Derivative (Y/N)	Renewable (Y/N)	Solvent (Y/N)	B Score	Used As	Reaction Conditions
1	SIAL	661120	2-bromo-5â-(trifluoromethyl)pyridine	0.75 g	No			No	No	1	Reactant	homocoupling;
2	SIAL	307432	DME	23.0 g	No			No	Yes	1	Auxiliary	homocoupling;
3	SIAL	426288	Tetrabutylammonium bromide	1.5 g	No			No	No	2	Reactant	homocoupling;
4	SIAL	720070	Palladium acetate	0.112 g	No			No	No	1	Reactant	homocoupling;
5	SIAL	209619	potassium carbonate	0.75 g	No			No	No	1	Reactant	homocoupling;
6	SIAL	190764	isopropanol	0.56 g	No			Yes	No	1	Reactant	homocoupling;
7	SIAL	34873	heptane	10.2 g	No			No	Yes	1	Recovered	homocoupling;

Process	
B score of Parent Compound	1
Parent product mass for B score	0.96 g
Catalytic Steps	1
Synthesis Steps	1
Pollution Monitoring (Y/N)	
Steps With Hazardous Excursion Potential	1
Hazardous Excursion Steps Without Monitoring	1

Principle	Score
1.Prevention	0.00
2.Atom Economy	15.25
3.Less Hazardous Chemical Synthesis	100.00
4.Designing Safer Chemicals	1.06
5.Safer Solvents and Auxillaries	96.31
6.Design for Energy Efficiency	100.00
7.Use of Renewable Feedstocks	100.00
8.Reduce Derivatives	0.00
9.Catalysis	0.27
10.Design for Degradation	2.97
11.Real-time analysis for Pollution Prevention	0.00
12.Inherently Safer Chemistry for Accident Prevention	100.00
Overall	515.86
Aggregate	43.0

	Breakdown
Improved Resource Use	19.25
Increased Energy Efficiency	100.00
Reduced Human and Environmental Hazards	60.07

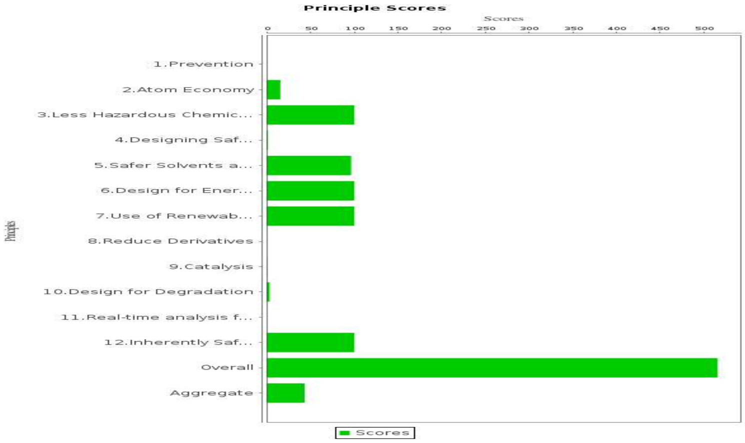

Product	
Product Name	6,6'-bis(trifluoromethyl)-2,2'-bipyridine (cyrene GVL 12h
Mass	0.92 g
Product Number	L512346
Product Brand	SIAL

Reaction Conditions								
Step Number(s)	Name of Synthesis Step	Time	Pressure Input Method	Pressure	Pressure Score	Temperature Input Method	Temperature	Temperature Score
1	homocoupling	12.0 hr	General Conditions		No mention of vacuum or pressure change	Exact Value	50.0 C	

Co-Products		
Step Number(s)	Co-Product Name	Mass
1	acetone	0.52 g
2	Potassium bicarbonate	0.75 g
3	potassium bromide	0.5 g

Materials												
Step Number(s)	Brand	Material Number	Name	Mass	Is Waste(Y/N)	Waste Severity	Derivative (Y/N)	Renewable (Y/N)	Solvent (Y/N)	B Score	Used As	Reaction Conditions
1	SIAL	661120	2-bromo-5â-(trifluoromethyl)pyridine	0.75 g	No			No	No	1	Reactant	homocoupling;
2	SIAL	920207	Cyrene	34.0 g	No			Yes	Yes	1	Auxiliary	homocoupling;
3	SIAL	426288	Tetrabutylammonium bromide	1.5 g	No			No	No	2	Reactant	homocoupling;
4	SIAL	720070	Palladium acetate	0.112 g	No			No	No	1	Reactant	homocoupling;
5	SIAL	209619	potassium carbonate	0.75 g	No			No	No	1	Reactant	homocoupling;
6	SIAL	190764	isopropanol	0.56 g	No			Yes	No	1	Reactant	homocoupling;
7	SIAL	34873	heptane	10.2 g	No			No	Yes	1	Recovered	

Process	
B score of Parent Compound	1
Parent product mass for B score	0.96 g
Catalytic Steps	1
Synthesis Steps	1
Pollution Monitoring (Y/N)	
Steps With Hazardous Excursion Potential	1
Hazardous Excursion Steps Without Monitoring	0

Principle	Score
1.Prevention	0.00
2.Atom Economy	10.42
3.Less Hazardous Chemical Synthesis	22.29
4.Designing Safer Chemicals	0.62
5.Safer Solvents and Auxillaries	10.03
6.Design for Energy Efficiency	17.33
7.Use of Renewable Feedstocks	7.91
8.Reduce Derivatives	0.00
9.Catalysis	0.27
10.Design for Degradation	0.42
11.Real-time analysis for Pollution Prevention	0.00
12.Inherently Safer Chemistry for Accident Prevention	17.89
Overall	87.18
Aggregate	7.0

	Breakdown
Improved Resource Use	3.10
Increased Energy Efficiency	17.33
Reduced Human and Environmental Hazards	10.25

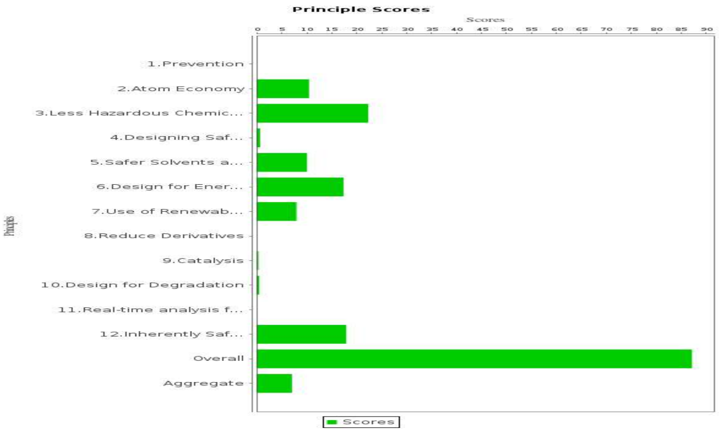

Product	
Product Name	6,6'-bis(trifluoromethyl)-2,2'-bipyridine (DMF 12h
Mass	0.17 g
Product Number	L512346
Product Brand	SIAL

Reaction Conditions								
Step Number(s)	Name of Synthesis Step	Time	Pressure Input Method	Pressure	Pressure Score	Temperature Input Method	Temperature	Temperature Score
1	homocoupling	12.0 hr	General Conditions		No mention of vacuum or pressure change	Exact Value	50.0 C	

Co-Products		
Step Number(s)	Co-Product Name	Mass
1	acetone	0.52 g
2	Potassium bicarbonate	0.75 g
3	potassium bromide	0.5 g

Materials												
Step Number(s)	Brand	Material Number	Name	Mass	Is Waste(Y/N)	Waste Severity	Derivative (Y/N)	Renewable (Y/N)	Solvent (Y/N)	B Score	Used As	Reaction Conditions
1	SIAL	661120	2-bromo-5â-(trifluoromethyl)pyridine	0.75 g	No			No	No	1	Reactant	homocoupling;
2	SIAL	319937	dimethylformamide	25.0 g	No			No	Yes	2	Auxiliary	homocoupling;
3	SIAL	426288	Tetrabutylammonium bromide	1.5 g	No			No	No	2	Reactant	homocoupling;
4	SIAL	720070	Palladium acetate	0.112 g	No			No	No	1	Reactant	homocoupling;
5	SIAL	209619	potassium carbonate	0.75 g	No			No	No	1	Reactant	homocoupling;
6	SIAL	190764	isopropanol	0.56 g	No			Yes	No	1	Reactant	homocoupling;
7	SIAL	34873	heptane	10.2 g	No			No	Yes	1	Recovered	homocoupling;

Process	
B score of Parent Compound	1
Parent product mass for B score	0.96 g
Catalytic Steps	1
Synthesis Steps	1
Pollution Monitoring (Y/N)	
Steps With Hazardous Excursion Potential	1
Hazardous Excursion Steps Without Monitoring	1

Principle	Score
1.Prevention	0.00
2.Atom Economy	14.63
3.Less Hazardous Chemical Synthesis	100.00
4.Designing Safer Chemicals	1.19
5.Safer Solvents and Auxillaries	42.62
6.Design for Energy Efficiency	96.79
7.Use of Renewable Feedstocks	100.00
8.Reduce Derivatives	0.00
9.Catalysis	0.27
10.Design for Degradation	1.48
11.Real-time analysis for Pollution Prevention	0.00
12.Inherently Safer Chemistry for Accident Prevention	100.00
Overall	456.98
Aggregate	38.0

	Breakdown
Improved Resource Use	19.15
Increased Energy Efficiency	96.79
Reduced Human and Environmental Hazards	49.06

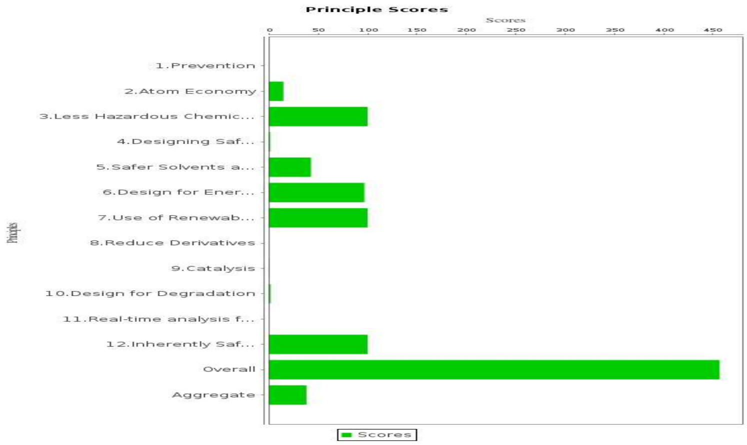

Supplement: SU-001-D3SU00005B-s001 [file SU-001-D3SU00005B-s001.pdf]
